# Different patterns, but no temporal decline in temperate forest soil meso‐ and macrofauna over the last decade

**DOI:** 10.1002/ecy.70246

**Published:** 2025-11-12

**Authors:** Melanie M. Pollierer, André Junggebauer, Sarah Bluhm, Melissa Jüds, Bernhard Klarner, Stefan Scheu

**Affiliations:** ^1^ J.F. Blumenbach Institute for Zoology and Anthropology University of Göttingen Göttingen Lower Saxony Germany; ^2^ Institute for Forest Protection Julius Kühn‐Institute (JKI) – Federal Research Center for Cultivated Plants Quedlinburg Saxony‐Anhalt Germany; ^3^ Centre of Biodiversity and Sustainable Land Use Göttingen Lower Saxony Germany

**Keywords:** abiotic drivers, belowground, Collembola, community stability, forest management, gamma diversity, global biodiversity decline, insurance hypothesis, Mesostigmata, Oribatida, portfolio effect, species richness

## Abstract

Global biodiversity loss is threatening ecosystem functioning and human well‐being. Arthropods above the ground have substantially decreased in abundance and diversity during the last 15–20 years. However, changes in belowground biodiversity, particularly in forests, received little attention. Here, we analyzed a comprehensive dataset of soil‐living meso‐ and macrofauna in forests differing in land‐use intensity within the framework of the open research platform “Biodiversity Exploratories” in Germany. The abundance of soil animal species was analyzed at 3‐year intervals, covering 12 years from 2008 to 2020. Neither species richness nor γ‐diversity of both soil meso‐ and macrofauna declined, suggesting contrasting dynamics of biodiversity above and below the ground. The density and diversity of soil mesofauna varied significantly between years within regions. These variations were closely related to the precipitation levels in the previous winter and during the sampling period. However, there was no consistent long‐term downward trend, as declines in some years were offset by full recoveries. Temporal trends of soil macrofauna taxa densities were inconsistent and depended on regions and forest management intensity. The stability of many soil taxa was related to effective diversity and asynchrony of species fluctuations, supporting the portfolio effect. However, variance ratios not different from null communities and a negative impact of temporal species turnover on stability suggest a minor influence of compensatory dynamics as predicted by the insurance hypothesis. Instead, strong abiotic control resulted in synchronous species dynamics. Species densities, particularly those of soil mesofauna, depended heavily on abiotic conditions, such as soil moisture. While influencing the density and richness of soil fauna and modulating the effects of precipitation, forest management did not directly affect the stability of soil fauna communities. While our findings demonstrate a remarkable resilience of soil animal communities in temperate German forests amidst ongoing biodiversity decline, they are based on a limited temporal window and forests in central Europe. As such, caution is needed when extrapolating these results to longer timescales or wider spatial scales. Nonetheless, our study provides valuable insights into the temporal dynamics of soil faunal density and diversity, and the key drivers underlying their community stability.

## INTRODUCTION

Understanding the patterns and drivers of species distributions is essential for mitigating the increasing global biodiversity decline, one of the major challenges of the Anthropocene (Cardoso et al., 2020; Dirzo et al., 2014; Pimm et al., 2014). Biodiversity loss is evident across a wide range of taxa, threatening ecosystem functioning (Cardinale et al., 2012; Oliver et al., 2015). In particular, the decline in terrestrial arthropod abundance and diversity has received considerable attention (Ceballos et al., 2015; Cowie et al., 2022; Hallmann et al., 2017; Van Klink et al., 2020; Wagner et al., 2021). However, most studies to date have focused on aboveground biodiversity, whereas studies on temporal variations of belowground biota are scarce (Guerra et al., 2020). This is particularly at odds with the fact that soils harbor more than half of the species on Earth, with about 30% of arthropod species living in soil (Anthony et al., 2023). Soil biota substantially contribute to important ecosystem functions such as decomposition, nutrient cycling, and carbon sequestration, thereby governing the productivity of terrestrial ecosystems (Bardgett & Van Der Putten, 2014; Moore et al., 2003; Wagg et al., 2014). Despite their importance, soil arthropods are often overlooked or only considered marginally in conservation management (Zeiss et al., 2022).

Aboveground arthropod declines have been linked to land‐use intensification (Asbeck et al., 2021; Seibold et al., 2019; Van Klink et al., 2020), which has also been identified as one of the major drivers of soil biodiversity decline (Geisen et al., 2019; Sünnemann et al., 2023; Tibbett et al., 2020). However, dynamics and drivers of biodiversity in soil differ from those above the ground (Cameron et al., 2019; Le Provost et al., 2021). Effects of land‐use change likely depend on management intensity, but also on the type of land‐use change and the type of ecosystem. In forests, soil animal communities likely are buffered against changes in management intensity and may be more influenced by abiotic drivers such as precipitation, temperature, and pH (Pollierer et al., 2021). However, existing studies mostly represent snapshots of soil animal biodiversity or are confined to certain taxonomic groups (Caruso et al., 2020; Junggebauer, Bluhm, et al., 2024; Kuznetsova, 2007), while studies exploring how management intensity and abiotic drivers affect forest soil animals over longer temporal scales are lacking (Guerra et al., 2020). Therefore, a comprehensive assessment of temporal changes in soil animal density and diversity in forests of different management intensity is urgently needed to evaluate and potentially mitigate long‐term biodiversity declines.

Biodiversity at the local scale (α‐diversity) presumably most rapidly responds to short‐term variations in abiotic drivers, whereas total diversity at the landscape scale (γ‐diversity) may undergo slower but more persistent changes due to land use or climate change, for example, as a consequence of habitat homogenization (Magurran, 2021; Zhang et al., 2014). Changes in diversity between habitats (β‐diversity) reflect the turnover in species composition among local communities and capture spatial heterogeneity and community differentiation across sites, thereby providing insight into how local dynamics scale up to shape regional biodiversity patterns. Temporal patterns may differ between soil meso‐ and macrofauna (Decaëns, 2010), as these groups are differentially affected by environmental drivers (Johnston & Sibly, 2020) and differ in their trophic/functional organization (Potapov et al., 2021; Turnbull et al., 2014). Due to small body size, short lifespans, and high sensitivity to desiccation, soil mesofauna may respond more rapidly to changing environmental conditions but may also recover more quickly due to higher reproductive rates (Bokhorst et al., 2012; Pollierer & Scheu, 2017). Macrofauna typically are long‐lived and can survive unfavorable conditions via dormant stages (Holmstrup, 2003).

Biodiversity is assumed to stabilize temporal dynamics of ecosystems (Campbell et al., 2011; Tilman et al., 2006). Community stability can be described by the inverse coefficient of variation (CV), which measures the relation between the temporal mean and the temporal variation of species (Hector et al., 2010). Stabilizing effects of biodiversity on community dynamics can arise through two non‐exclusive mechanisms. First, the *portfolio effect* posits that diverse communities exhibit reduced variability through statistical averaging of asynchronous species fluctuations, akin to risk‐spreading in financial portfolios (Doak et al., 1998; Schindler et al., 2015). This mechanism depends on effective diversity (i.e., the number and evenness of species) and does not require ecological interactions. Second, the *insurance hypothesis* emphasizes compensatory dynamics driven by functional differences or biotic interactions, where declines of some species are offset by others, stabilizing aggregate abundance (Loreau & de Mazancourt, 2013; Yachi & Loreau, 1999). While both mechanisms rely on species asynchrony, the portfolio effect is a statistical outcome, whereas the insurance hypothesis implies ecological processes. Additionally, higher population densities may stabilize communities by buffering stochasticity independently of diversity (Rooney & McCann, 2012). For aboveground arthropods and vertebrates in forests and grasslands, the insurance hypothesis has been identified as a key mechanism promoting stability (Blüthgen et al., 2016). This may also hold for soil animal communities but may not be sufficient to explain patterns of stability (Caruso et al., 2020; Junggebauer, Bluhm, et al., 2024).

Here, we present the first comprehensive assessment of long‐term temporal variations in soil fauna density and diversity in forests, including major taxa of mesofauna (Oribatida, Collembola, and Mesostigmata) and macrofauna (Lumbricidae, Isopoda, Diplopoda, Chilopoda, Coleoptera, and Araneae). Within the Biodiversity Exploratories—a large‐scale, long‐term research platform across three German regions—we collected soil fauna data over 12 years at 3‐year intervals, and related them to environmental drivers such as forest management, precipitation, and microbial biomass. We asked the following questions: (1) Do soil fauna density, species richness, and γ‐diversity show declining trends similar to those of aboveground invertebrates? (2) Are temporal changes in soil fauna communities linked to climatic variation and forest management? (3) What mechanisms govern the stability of these communities? We hypothesized that (1) temporal variations in density and diversity of soil meso‐ and macrofauna largely depend on abiotic drivers, such as precipitation and temperature, and less on forest management. Further, (2) temporal variations are more pronounced in soil mesofauna due to shorter lifespans, higher rates of reproduction, and higher sensitivity to abiotic fluctuations than in soil macrofauna. (3) In line with the insurance hypothesis, stability, as reflected by the inverse CV, is influenced by asynchronous species fluctuations of both meso‐ and macrofauna. Finally, (4) there is no consistent temporal decline in density and diversity of soil fauna due to the buffering effects of the soil habitat.

## MATERIALS AND METHODS

### Study sites and environmental variables

The study sites were part of the “Biodiversity Exploratories,” an open platform for biodiversity and ecosystem research (www.biodiversity-exploratories.de; Fischer et al., 2010), covering three regions in northern, central, and southern Germany, that is, Schorfheide–Chorin (northern), the Hainich–Dün (central), and the Swabian Alb (southern). Temperature and precipitation were measured at the plot level at 10‐min resolution and aggregated to the month (Biodiversity Exploratories Instrumentation Project; BExIS dataset ID 24766; Wöllauer et al., 2021). We calculated mean values for winter (December to February) and spring (March to May) and assessed microbial biomass (*C*_mic_) in leaf litter and soil in each plot and year, parallel to the sampling of soil animals. For details on study sites and environmental variables, please refer to Appendix S1: Section S1. Data on environmental variables are archived in Dryad (Pollierer et al., 2025a).

### Sampling, extraction, and determination of soil animals

In each sampling year, samples were collected between April and June, progressing sequentially from the northern to the southern region to account for regional differences in the start of the growing season. This was done at 3‐year intervals from 2008 to 2020, resulting in five sampling points. While this sampling design does not capture short‐term fluctuations, particularly in mesofauna populations, it is appropriate for assessing long‐term trends in density and diversity. In each of the 48 forests sampled (with 16 forests in each of the three regions, at least 200 m apart), samples were taken in a 5 × 5 m subplot within a single 100 × 100 m gridplot as described in Klarner et al. (2014). From this subplot, one 5‐cm‐diameter soil core was collected for the extraction of mesofauna, and one 20‐cm‐diameter core for the extraction of macrofauna. We separated the litter layer (variable thickness) and the top 5 cm of the soil underneath for extracting meso‐ and macrofauna by heat (Macfadyen, 1961). Individuals from the two layers were pooled for statistical analyses. Lumbricidae were sampled from an area of 0.25 m^2^ from each subplot using a combination of hand sorting and extraction with mustard solution. First, the litter layer was removed and checked manually for earthworm specimens. Then, a mustard solution consisting of 100 g of mustard powder (Semen Sinapis plv.; CAELO, Cesar & Loretz GmBH, Hilden, Germany) dissolved in 10 L of water was applied to the soil in two steps with initially 5 L and another 5 L after 15 min (Eisenhauer et al., 2008). Emerging Lumbricidae were hand‐collected for a total of 30 min. All animals were stored in 70% ethanol until identification. Mesofauna (Oribatida, Collembola, and Mesostigmata) and macrofauna taxa (Isopoda, Diplopoda, Lumbricidae, and Chilopoda) were identified to species level using appropriate keys (Eason, 1964; Hopkin, 2007; Karg, 1989, 1993; Krantz & Ainscough, 1990; Oliver & Meechan, 1993; Schubart, 1934; Sims, 1988; Weigmann, 2006). Araneae and Coleoptera were only sorted at the taxon level and were not considered in the analyses of diversity. Soil mesofauna and macrofauna densities at the species/group level from 2008 to 2020 are available in Dryad (Pollierer et al., 2025a).

### Metrics of diversity and stability

Species richness was calculated as the number of species per soil core (or per 0.25 m^2^ for Lumbricidae). Effective diversity was estimated as the exponential of Shannon entropy ($e^{H^{\prime }}$), where $H^{\prime }=\sum_{i=1}^{n}p_{i}\;\log\left(p_{i}\right)$, with *p*_*i*_ as the relative abundance of species *i* (Jost, 2006).

Beta diversity was assessed only for mesofauna, due to higher species richness and more consistent sampling, which enabled more robust estimates of species turnover compared to macrofauna. Total β diversity (BD_total_) was calculated as the total variance in a community matrix (Legendre & De Cáceres, 2013) using the *beta.div*() function in the *adespatial* package (Dray et al., 2019), with Hellinger‐transformed abundance data. BD_total_ was computed per taxon, region, and year, with significance assessed via 999 permutations. We also extracted local contributions to β diversity (LCBD), that is, the compositional uniqueness of each site, for each plot and year, using the mean LCBD across years as a supplementary predictor of temporal stability (Appendix S1: Section S2). Due to collinearity with effective diversity, LCBD was not included in the main stability models.

Gamma diversity was estimated annually for mesofauna (Oribatida, Collembola, Mesostigmata) and for macrofauna (Isopoda, Diplopoda, Lumbricidae, Chilopoda) using coverage‐based rarefaction and extrapolation (Chao & Jost, 2012) implemented in the *iNEXT* package (Hsieh et al., 2016), based on incidence data and focusing on *q* = 0, corresponding to species richness. Diversity was extrapolated to double the smallest reference sample size, with 95% CI estimated from 1000 bootstraps (Appendix S1: Figures S3 and S4; Table S2).

Community stability was measured as the inverse CV: S=μσ, where μ and σ are the temporal mean and SD of total abundance per site (Tilman, 1999; Tilman et al., 2006).

To assess species asynchrony, we calculated the variance ratio (VR), comparing community‐level variance to the sum of species‐level variances: VR=VarC∑i=1nVarxi, where *C* is the total community abundance and $x_{i}$ denotes the abundance of species *i* (Schluter, 1984). VR <1 indicates compensatory (asynchronous) dynamics; VR >1 indicates synchronous fluctuations. Significance was assessed using 10,000 cyclic shift permutations. We also calculated species synchrony (η) following Gross et al. (2014), a richness‐insensitive metric. For clarity, we report synchrony as “asynchrony” (1 − η), with higher values reflecting greater species compensation. Stability, variance ratio, and synchrony were calculated across all 48 forest sites and five sampling dates using the *codyn* package (Hallett et al., 2016) for mesofauna and for Chilopoda and Lumbricidae. Isopoda and Diplopoda were excluded due to low species richness and patchy distribution.

To test whether communities met the statistical preconditions for the portfolio effect, we estimated the scaling exponent *z* from Taylor's power law (σ^2^ = *c*μ^*z*^; Doak et al., 1998), including region × mean abundance interactions in the models. For all major taxa, *z* ranged from 1.41 to 1.72 (Appendix S1: Figures S5 and S6), indicating that variance increased less than quadratically with the mean. This suggests that statistical averaging among species could reduce temporal variability, a necessary condition for the portfolio effect (Tilman et al., 1998). Regions with steeper slopes (higher *z*) may reflect more synchronous species dynamics, potentially driven by shared abiotic influences and reduced compensatory buffering (Zhao et al., 2019).

### Statistical analyses

All analyses were conducted in R v. 4.3.2 (R Core Team, 2020). To assess temporal trends in soil fauna density and richness, we fitted mixed‐effects models with year, taxon, region, silvicultural management intensity indicator (SMI; Schall & Ammer, 2013), precipitation (winter and spring), their interactions, and litter microbial biomass as fixed effects. Plot was included as a random factor. Continuous predictors were standardized, and abiotic correlations were explored via principal components analysis (PCA) (Appendix S1: Figure S7). Precipitation was used instead of temperature based on superior model performance (*DHARMa* and *performance* packages; Hartig, 2022; Lüdecke et al., 2021). Response distributions were assessed using *fitdistrplus* (Delignette‐Muller et al., 2015). Count data (mesofauna density, macrofauna density, and richness) were modeled with *glmmTMB* (Brooks et al., 2023; negative binomial distribution) and mesofauna richness with *lme4* (Bates et al., 2015; Gaussian error). Predicted trends and 95% CI were derived using the *effects* and *emmeans* packages (Fox et al., 2016; Lenth et al., 2023), with significance tested via Wald tests. To identify drivers of stability, we fitted linear models per taxon using log‐transformed stability as the response. Predictor variables were added sequentially in theory‐driven order: (1) total abundance and region, (2) effective diversity, (3) asynchrony, and (4) SMI. In addition, we tested mean LCBD as a separate predictor of stability in a supplementary analysis due to its strong collinearity with other variables. The code used for the analyses and figure generation is archived in Zenodo (Pollierer et al., 2025b).

## RESULTS

### Variations in density and diversity of soil mesofauna

**FIGURE 1:**
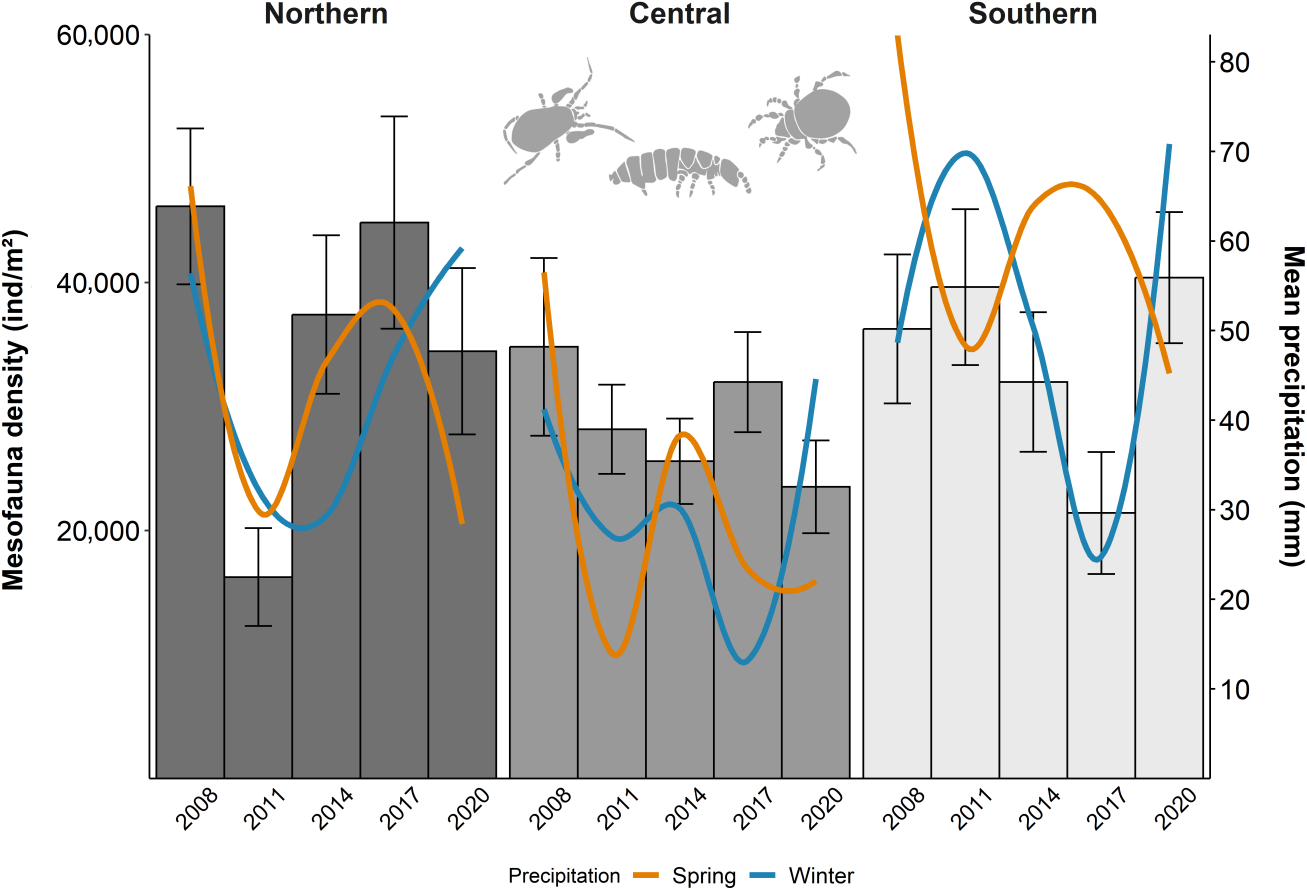
Mean density of mesofauna, including Oribatida, Collembola, and Mesostigmata (±SE) in northern, central, and southern regions in Germany between 2008 and 2020, evaluated at 3‐year intervals. Mean precipitation during the preceding winter months (December, January, February) for each region is plotted as a blue smoothed line, and mean precipitation during the spring months (March, April, May) at the time of sampling is plotted as an orange smoothed line. Mesofauna silhouettes (owned by Stefan Scheu) were illustrated by Svenja Meyer.

**FIGURE 2:**
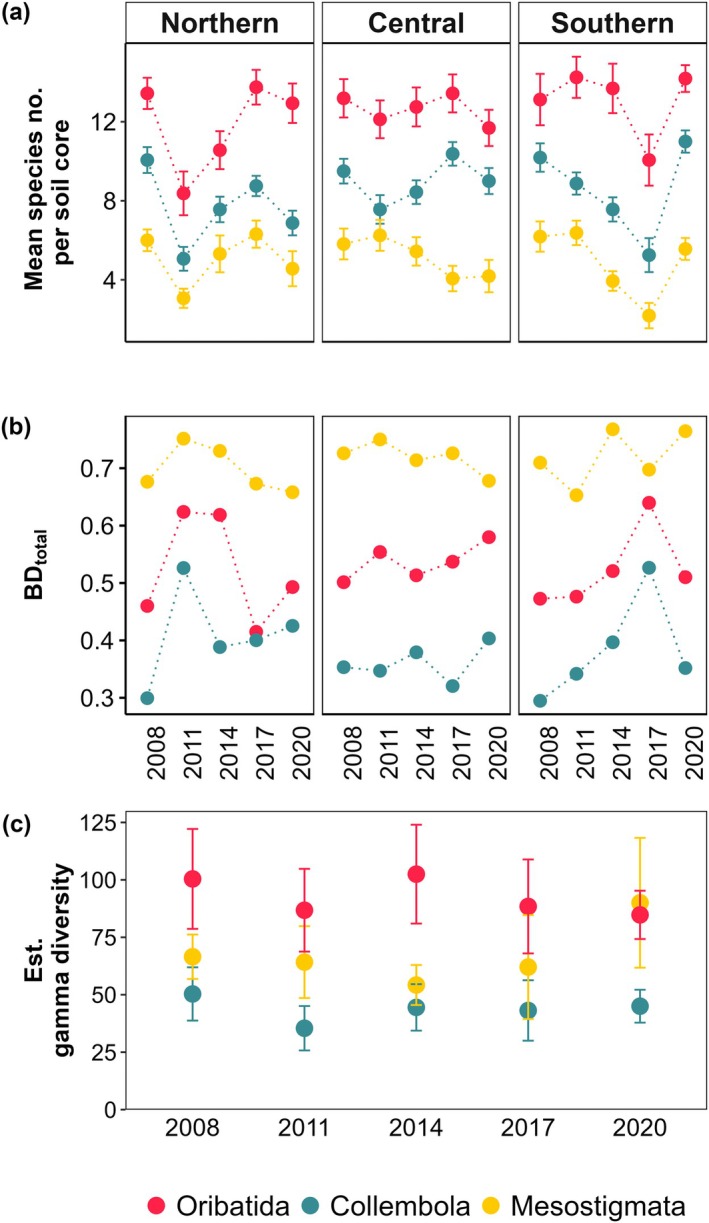
Species richness (a), β‐diversity (BD_total_; b), and estimated γ‐diversity (c) of the mesofauna taxa Oribatida (red), Collembola (blue), and Mesostigmata (yellow) sampled at 3‐year intervals from 2008 to 2020 in the northern, central, and southern regions of Germany.

Total density and species richness of soil mesofauna across regions were similar between years; however, within regions they varied significantly between years, with significantly lower densities and species richness in the northern sites in 2011 and in the southern sites in 2017 (year × region: *F*_2,660_ = 10.43, *p* < 0.0001 and *F*_2,661_ = 3.96, *p* = 0.019, respectively; Figures 1 and 2a, Appendix S1: Table S3). Density and species richness were significantly affected by both the precipitation of the preceding winter months (region × precipitation winter: *F*_2,653_ = 16.91, *p* < 0.001 and *F*_2,663_ = 10.14, *p* < 0.001, for density and richness, respectively) and the precipitation in spring, that is, at the time of sampling (region × precipitation spring: *F*_2,646_ = 16.42, *p* < 0.001 and *F*_2,648_ = 5.17, *p* = 0.006, for density and richness, respectively; Appendix S1: Table S3), with region‐specific responses. In the northern region, spring precipitation had the strongest effect, while in the southern region, winter precipitation was more influential. No significant effects were observed in the central region (Figures 1 and 2a; Appendix S1: Figure S8; Table S4). Forest management intensity (SMI) positively influenced mesofauna densities (*F*_1,108_ = 5.95, *p* = 0.016; Appendix S1: Figure S9) and interacted with precipitation (SMI × precipitation spring: *F*_1,651_ = 4.81, *p* = 0.029; region × SMI × precipitation winter: *F*_2,657_ = 3.74, *p* = 0.024). Positive effects of spring precipitation were more pronounced under higher management intensity, while winter precipitation generally had a positive effect across regions, with some variation in strength. Mesofauna species richness was influenced by forest management intensity, but effects varied by taxon and region (taxon × region × SMI: *F*_4,627_ = 2.77, *p* = 0.027; Appendix S1: Table S3). No consistent patterns emerged within individual taxon–region combinations. Microbial biomass in leaf litter neither significantly influenced density nor species richness of soil mesofauna (Appendix S1: Table S3).

Density and species richness of mesofauna differed between the three taxa studied (*F*_2,623_ = 448.03, *p* < 0.001 and *F*_2,627_ = 320.25, *p* < 0.001, respectively; Appendix S1: Table S3), with the density of Oribatida (52,800 ± 51,893 ind/m^2^, overall mean ± SD) and Collembola (39,473 ± 29,720 ind/m^2^) being much higher than that of Mesostigmata (6483 ± 6175 ind/m^2^). Species richness was also highest in Oribatida (13.5 ± 4.3 species per soil core, overall mean ± SD), intermediate in Collembola (8.4 ± 3.0 species per soil core), and lowest in Mesostigmata (5.0 ± 3.0 species per soil core), but differences in richness between taxa were not as pronounced as those in density. In addition, the density of mesofauna differed significantly between regions (*F*_2,88_ = 4.04, *p* = 0.021), with higher density in the northern (35,817 ± 46,244 ind/m^2^, mean ± SD) than in the southern (33,931 ± 39,418 ind/m^2^) and central region (28,852 ± 31,954 ind/m^2^).

Annual variations in mesofauna density and diversity were similar among taxa (Appendix S1: Table S3). Gamma‐diversity across the three regions was highest in Oribatida, followed by Mesostigmata and Collembola, with the exception of 2020, where γ‐diversity was similarly high in Mesostigmata and Oribatida. While species richness was higher in Collembola than in Mesostigmata (Figure 2a), γ‐diversity showed the opposite pattern, being higher in Mesostigmata than in Collembola (Figure 2c). Beta diversity (BD_total_) was highest in Mesostigmata, followed by Oribatida and Collembola (Figure 2b). Notably, BD_total_ peaked in years when both densities and species richness were lowest, indicating an inverse relationship with these metrics. Within the 12‐year time span from 2008 until 2020, γ‐diversity of Oribatida, Collembola, and Mesostigmata neither differed significantly between years nor showed an overall decline across the three regions in Germany (Figure 2c).

### Variations in density and diversity of soil macrofauna

**FIGURE 3:**
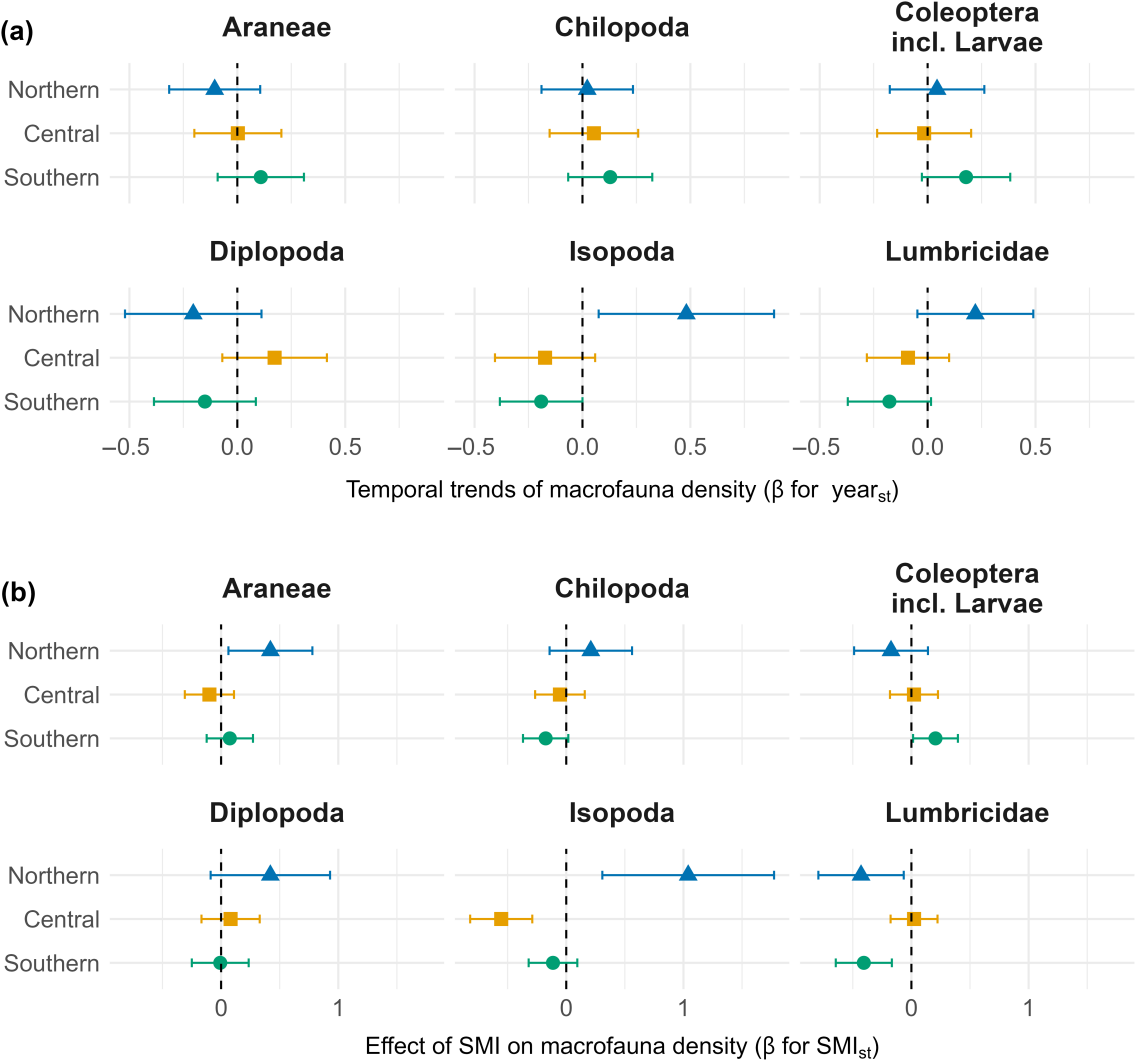
Estimated effects of (a) year of sampling and (b) silvicultural management intensity (SMI) on macrofauna density (abundance per soil core) across taxonomic groups in three regions of Germany (northern, central, southern). Effect sizes (β values) are derived from a generalized linear mixed‐effects model (GLMM) with a negative binomial distribution (see Appendix S1: Table S5). Points and error bars represent estimated standardized model coefficients and 95% CI, respectively, for each taxon within each region. Positive values indicate increasing trends in density with increasing year or SMI, while negative values indicate decreasing trends. Statistical details for each estimate (SE, *z*‐ratio, *p*‐value) are provided in Appendix S1: Tables S6 (year) and S7 (SMI).

Neither species richness nor γ‐diversity of the soil macrofauna was significantly influenced by the sampling year or its interactions, which in γ‐diversity was due to large variability (Appendix S1: Table S5; Figure S10). However, the density of macrofauna differed between years depending on taxon and region (year × taxon × region: χ^2^ (10) = 25.12, *p* = 0.005; Figure 3a; Appendix S1: Figure S11; Table S6); in addition, these differences were influenced by forest management as indicated by the SMI (year × taxon × region × SMI: χ^2^ (10) = 18.48, *p* = 0.047; Appendix S1: Table S5; Figure S12). Overall, temporal trends of macrofauna densities in the different regions were mostly neutral; only Isopoda had a significantly positive trend in the northern region, mainly at high and intermediate SMI, and a negative trend in the southern region, but only at low SMI. In the central region, Isopoda had a negative temporal trend at low SMI, but a positive trend at high SMI (Appendix S1: Figure S12). Species richness of soil macrofauna differed significantly between taxa depending on region (taxon × region: χ^2^ (6) = 28.98, *p* < 0.001; Appendix S1: Table S5; Figure S13), with generally lower macrofauna richness in the northern region. Across years, density, and species richness of macrofauna taxa were significantly influenced by forest management, with effects depending on region (taxon × region × SMI: χ^2^ (10) = 52.42, *p* < 0.001 and χ^2^ (6) = 19.10, *p* = 0.004, respectively; Figure 3b, Appendix S1: Tables S5, S7, and S8; Figure S14). In the northern region, densities of Araneae and Isopoda were positively influenced by forest management, whereas Lumbricidae densities were negatively influenced by forest management. In the central region, densities of most taxa did not significantly respond to management intensity, except for Isopoda, which were negatively influenced by higher SMI. In the southern region, densities of Lumbricidae and in trend of Chilopoda were negatively influenced by forest management, whereas Coleoptera and their larvae were positively influenced. Species richness of Chilopoda and in the trend of Isopoda was negatively influenced by forest management in the central region, while that of Lumbricidae was negatively influenced in the northern and southern regions (Appendix S1: Table S8; Figure S14).

### Patterns and causes of community stability

**FIGURE 4:**
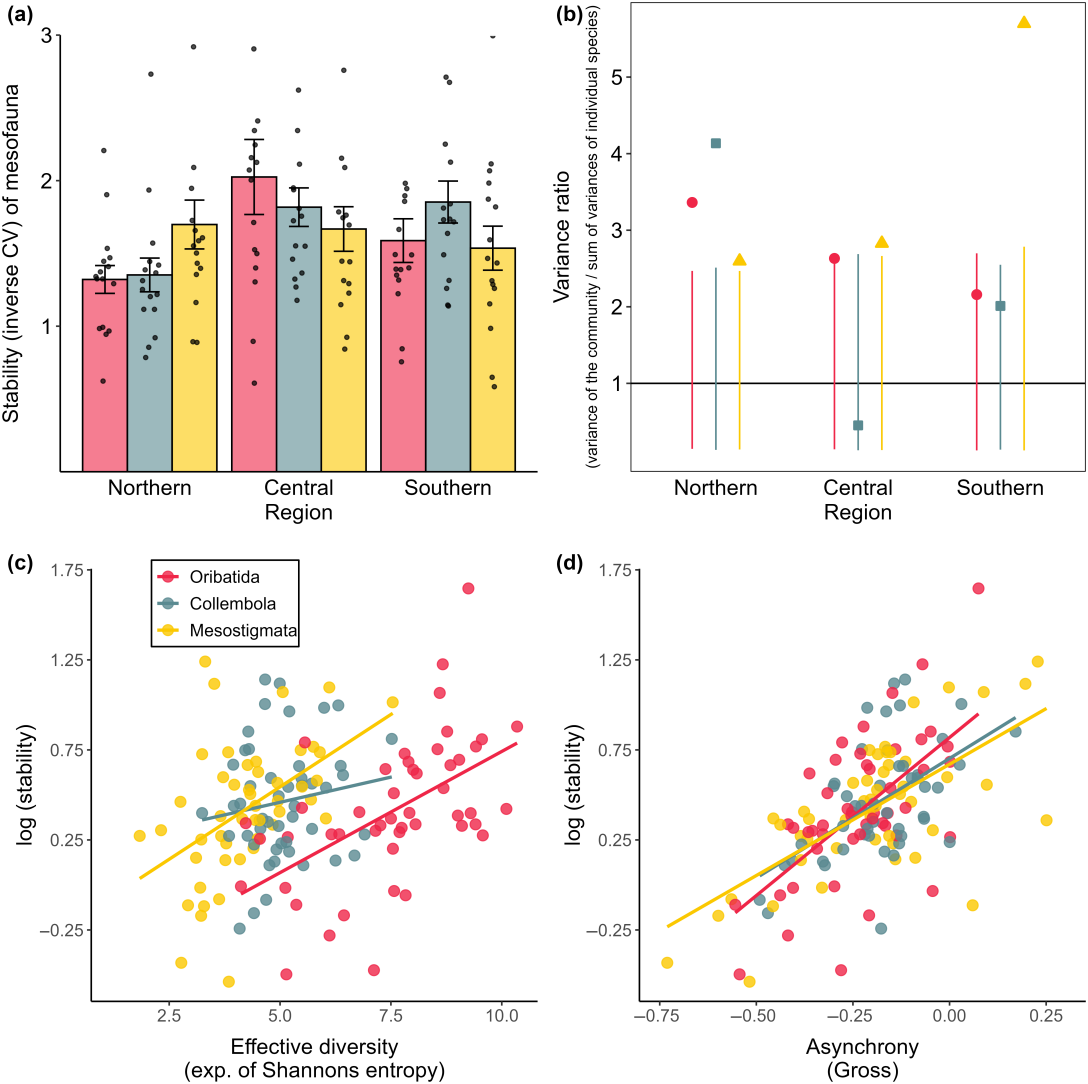
(a) Stability (inverse CV; means ± SE and raw data points) of the mesofauna taxa Oribatida (red), Collembola (blue), and Mesostigmata (yellow) in the studied northern, central, and southern regions in Germany. The variance ratio (b), that is, the variance of the total density of individuals divided by the sum of variance of individual species, indicates whether species abundances within communities of Oribatida (red dots), Collembola (blue squares), and Mesostigmata (yellow triangles) co‐vary mostly positively (synchronous fluctuations, variance ratio > 1) or negatively (asynchronous fluctuations, variance ratio < 1). The null mean calculated for each community approximated one and is indicated by the black solid line; colored lines indicate the CI of the null models. Points outside the lines are significantly different from random. (c) and (d) The relation of stability of mesofauna communities with effective diversity and asynchrony.

The stability of mesofauna communities did not differ between Oribatida, Collembola, and Mesostigmata, but differed significantly between regions (*F*_2,120_ = 4.50, *p* = 0.013), with the lowest stability in the northern region (1.46 ± 0.54), intermediate stability in the southern region (1.66 ± 0.60) and the highest stability in the central region (1.84 ± 0.76). Regional differences also marginally depended on taxa (taxon × region: *F*_4,120_ = 2.16, *p* = 0.078, Figure 4a; Appendix S1: Table S9). While stability was similar across regions in Mesostigmata, it was lower in Oribatida and Collembola in the northern regions compared to the other regions. In addition, mean precipitation in winter significantly influenced stability, with effects depending on region (region × precipitation winter: *F*_2,120_ = 5.28, *p* = 0.006; Appendix S1: Table S9; Figure S15). While precipitation positively affected stability in the central region, it had no effect in the northern region and a slightly negative effect in the southern region. The stability of Oribatida and Mesostigmata was significantly positively influenced by their effective diversity (*F*_1,33_ = 16.44 and *F*_1,33_ = 25.54, respectively; both *p* < 0.001; Figure 4c; Appendix S1: Table S10). In all three mesofauna taxa, stability also increased significantly with higher levels of species asynchrony (*F*_1,33_ = 17.55, *F*_1,33_ = 12.56, and *F*_1,33_ = 55.85 for Oribatida, Collembola, and Mesostigmata, respectively; all *p* ≤ 0.001; Figure 4d). However, variance ratios greater than one—particularly in the northern region—and mostly negative values of species asynchrony (Figure 4b,d) indicate that species tended to fluctuate synchronously. Consistent with this, Taylor's power law models revealed steeper variance–mean relationships (i.e., higher *z* values) in the northern region, also indicating greater population synchrony compared to the central and southern regions (Appendix S1: Figure S5). In addition, mean LCBD was negatively associated with stability, particularly in Mesostigmata (Appendix S1: Section S2; Figure S1), indicating that higher compositional turnover tended to coincide with reduced community stability rather than reflecting stabilizing compensatory dynamics.

**FIGURE 5:**
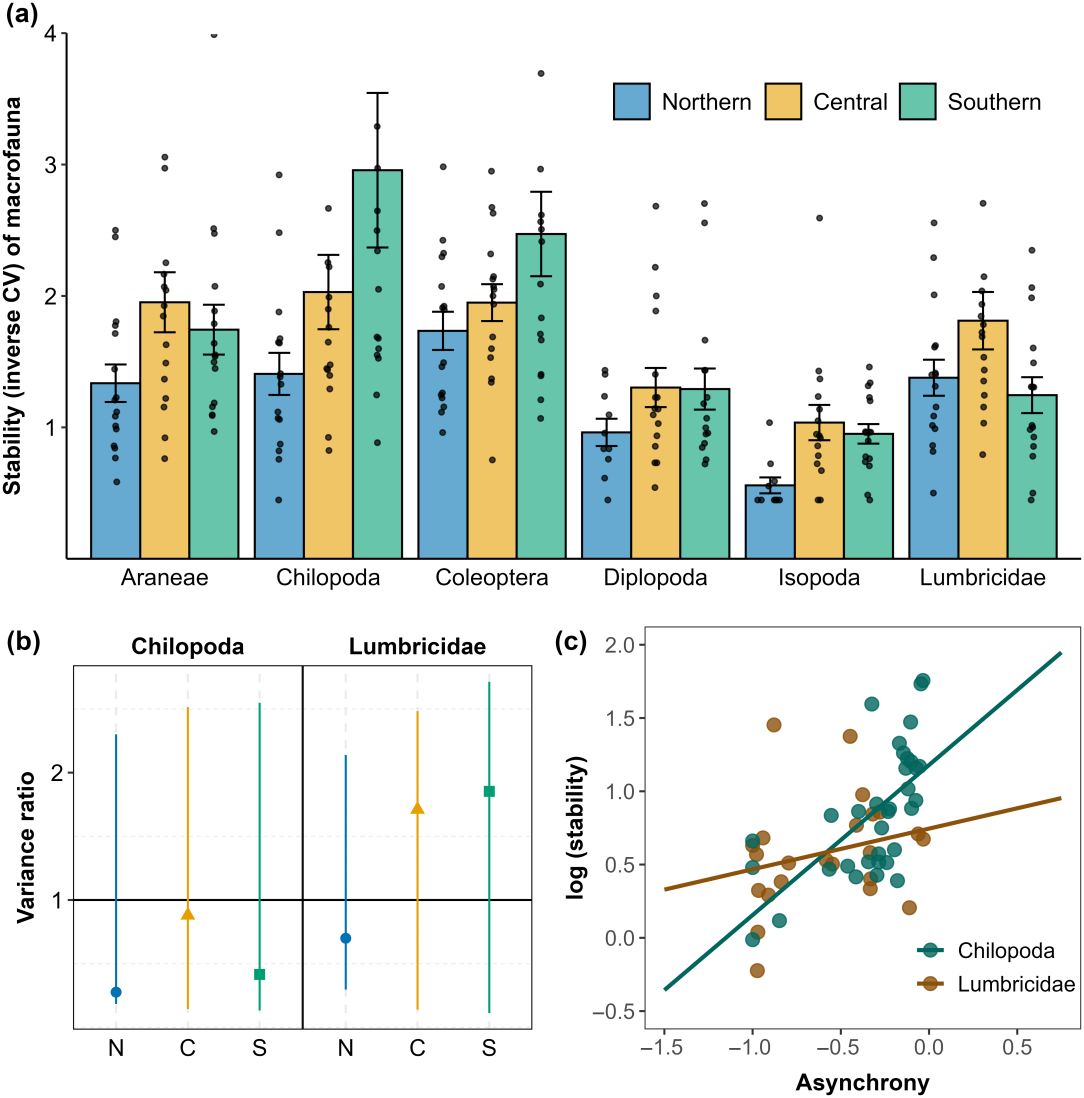
(a) Stability (inverse CV; means ± SE and raw data points) of macrofauna taxa in the studied northern, central, and southern regions of Germany, and (b) the variance ratio, that is, the variance of the total density of individuals divided by the sum of variances of individual species of Chilopoda and Lumbricidae communities in the studied northern, central, and southern regions in Germany. The null mean calculated for each community approximated one and is indicated by the black solid line; colored lines indicate the CI of the null models. (c) Stability of Chilopoda and Lumbricidae communities as influenced by asynchrony; each dot represents one forest site.

Stability of macrofauna differed between taxa, with differences depending on region (taxon × region: *F*_10,232_ = 1.94, *p* = 0.040; Figure 5a; Appendix S1: Table S9). In most taxa, stability was lower in the northern region, except for Lumbricidae, with the highest stability in the central region and similarly low stability in the northern and southern regions. In Chilopoda, stability was significantly influenced by asynchrony (*F*_1,22_ = 16.91, *p* < 0.001; Figure 5c; Appendix S1: Table S11); however, a variance ratio not different from null communities (Figure 5b) and mostly negative values of asynchrony indicated mainly synchronous fluctuations. By contrast, the stability of Lumbricidae communities did not depend on any of the analyzed variables.

## DISCUSSION

We presented a comprehensive long‐term assessment of the density and diversity of soil fauna along a gradient of forest management intensity in Germany, spanning 12 years from 2008 to 2020. Across the three regions studied, γ‐diversity neither declined significantly in soil mesofauna nor in soil macrofauna, suggesting that belowground biodiversity of temperate forests in Germany does not follow the declining pattern reported for aboveground biodiversity (Seibold et al., 2019). Regional temporal variation in soil mesofauna was primarily driven by precipitation, while macrofauna diversity showed little temporal change, and density trends were inconsistent across taxa. Strong abiotic control led to largely synchronous species fluctuations, especially in mesofauna, as indicated by variance ratios not significantly different from null expectations. Despite this, community stability was positively related to asynchrony, suggesting that even low levels of compensatory dynamics can enhance stability. These patterns are consistent with the portfolio effect via statistical averaging but provide limited support for the insurance hypothesis based on strong compensatory responses.

### Temporal variation of density and diversity of soil fauna

Temporal variation of mesofauna densities and species richness was more pronounced in the northern and southern regions, where they were linked to patterns of precipitation during the time of sampling and in the preceding winter, respectively, in part supporting our first hypothesis. The lack of correlation with climatic variables in the central region is likely due to its deep clay‐rich soils and a pronounced litter layer, which buffers against variations in precipitation. By contrast, the shallow rendzina soils in the southern region and the sandy soils in the northern region, featuring low water‐holding capacity, are more prone to desiccation. Precipitation and corresponding soil moisture have been identified as important drivers of soil animal density and diversity in different ecosystems (Wu et al., 2023; Zhang et al., 2014); however, the effects of precipitation are often context dependent (Meyer et al., 2021). More pronounced effects of spring precipitation at higher management intensity on mesofauna density were potentially due to higher proportions of asexual species in the most intensively managed coniferous forests, able to respond more quickly to favorable environmental conditions (Bluhm et al., 2016; Pollierer & Scheu, 2017). Forest management intensity generally was associated with increased soil mesofauna density, potentially due to thick organic layers in coniferous forests providing large habitat space (Bluhm et al., 2016; Erdmann et al., 2012), whereas effects on species richness depended on taxon and region but were generally not pronounced. Microbial biomass in leaf litter, representing a proxy for potential food resources of detritivore soil fauna, did not significantly influence the density and diversity of mesofauna; potentially, the often‐observed poor correlation between microbial biomass in leaf litter and soil fauna is due to foraging at microsites rather than consumption of microorganisms in bulk litter or soil (Ferlian et al., 2015). However, it may also support our conclusion that abiotic, rather than biotic drivers, and not resources, predominantly control soil fauna density and diversity in temperate forests. Notably, despite significant differences in density between mesofauna taxa, with six‐ and even eightfold higher densities of Collembola and Oribatida compared to Mesostigmata, respectively, temporal variations of both density and species richness were similar among mesofauna taxa, supporting the importance of abiotic control. In contrast to density, species richness of Mesostigmata was not considerably lower than that of the other taxa, with only 1.7‐ and 2.7‐times higher species richness in Collembola and Oribatida. Across all regions, γ‐diversity of Mesostigmata was even higher than that of Collembola due to higher total species numbers and higher turnover, that is, higher beta‐diversity.

In soil macrofauna, neither γ‐diversity nor species richness showed any significant temporal variation. The densities of the macrofauna followed different temporal trends, which were only significant for a few taxa and regions, and were impacted by forest management intensity. Trends at high management intensity were often positive, for example, for Chilopoda, Diplopoda, and Isopoda in the central region, and for Coleoptera in the southern region, potentially due to differences in forest dynamics at different management intensities. Despite SMI decreasing in most forests over the recorded period, the most intensively managed forests typically were young forests, in which habitat structure, resource availability, and microclimate likely improved during maturation, whereas forests of low management intensity typically were older and therefore likely changed less during the study period. Forest management intensity also directly affected macrofauna densities in taxon‐ and region‐specific ways—with negative effects on Chilopoda in the southern region, on Lumbricidae in the northern and southern regions, and on Isopoda in the central region, likely due to altered litter quality and soil pH in intensively managed coniferous forests (Pollierer et al., 2021). By contrast, positive effects were observed for Isopoda and Araneae in the northern region and on Coleoptera in the southern region, potentially due to higher structural complexity resulting from higher amounts of leaf litter in more strongly managed forests. As densities of macrofauna taxa did not significantly depend on precipitation or temperature, biotic interactions, such as resource availability or predation, may better explain their temporal dynamics. Species richness of macrofauna was generally lowest in the northern region, presumably due to the above‐mentioned differences in soil type and harsh (continental) climatic conditions. In Diplopoda and Lumbricidae, it was significantly negatively influenced by forest management in the central region, and in Chilopoda in the northern and southern regions, likely for similar reasons as for the decline in densities. Despite somewhat lower γ‐diversity of soil macrofauna in 2014, differences between years were not significant, and species richness did not show significantly decreasing trends. However, densities of some macrofauna taxa, such as Isopoda and Lumbricidae, decreased over the observed timespan, especially in the southern region and at lower management intensity, indicating that macrofauna density may react sensitively to changing environmental conditions. Nevertheless, changes were not uniform and confined to few taxa, as also evident in nonsignificant changes in overall density, suggesting that, across the studied regions, soil macrofauna populations are relatively stable and, like mesofauna, do not follow an overall declining trend, at least not over the last decade.

### Drivers of stability in soil meso‐ and macrofauna

Stability of mesofauna differed between regions, with lower stability of Oribatida and Collembola in the northern region, likely due to harsh climatic conditions and higher and more synchronous fluctuations in density as indicated by significantly positive variance ratios, presumably mainly of parthenogenetic species (Pollierer & Scheu, 2017). In the central region, stability was positively correlated with mean precipitation in winter, with more favorable environmental conditions likely resulting in lower fluctuations of species densities. In Oribatida and Mesostigmata, effective diversity was a significant driver of stability, supporting the role of the portfolio effect, that is, statistical averaging of stochastic fluctuations of species densities, in maintaining community stability (Doak et al., 1998; Tilman et al., 1998). In Collembola, effective diversity only marginally contributed to stability, and this depended on region, with positive correlations only in the southern and northern regions. Although variance ratios indicated predominantly synchronous community dynamics, we found that even slight increases in species asynchrony were significantly associated with higher community stability in all mesofauna taxa. This further supports that statistical averaging of partially independent species fluctuations, that is, the portfolio effect, drives stability of soil mesofauna, whereas biotic compensation as posed by the insurance hypothesis is of minor importance, particularly in the northern and southern regions. By contrast, in relatively stable environments such as in the central region, biotic stability mechanisms may become more important (Hallett et al., 2014). Synchronous fluctuations were likely caused by abiotic drivers such as precipitation, which we identified as one of the main drivers of temporal fluctuations. The importance of abiotic environmental versus biotic, density‐dependent drivers of community dynamics is debated controversially (Shorrocks & Sevenster, 1995; Wilson & Lundberg, 2006); however, a number of studies suggest that community dynamics are often dominated by density‐independent abiotic drivers and that abundances of many taxa tend to covary positively (Houlahan et al., 2007; Mutshinda et al., 2009). In line with this, we found that community uniqueness over time, as measured by mean LCBD, was negatively associated with temporal stability, particularly in Mesostigmata. This suggests that plots with higher temporal turnover in species composition tended to be less stable, further indicating that high β‐diversity may not reflect stabilizing compensatory dynamics but rather the destabilizing influence of environmental fluctuations. Supporting this interpretation, total β‐diversity varied inversely with species richness and density across years and regions, with higher community turnover occurring in years of reduced mesofauna abundance and diversity. These patterns are consistent with the idea that strong abiotic forcing, particularly precipitation, drives synchronous responses across species, undermining the potential for biotic compensation and instead amplifying turnover. Together, these results highlight that statistical averaging (i.e., the portfolio effect) stabilizes communities despite limited asynchrony, whereas the destabilizing role of environmental variability becomes apparent in more compositionally dynamic (i.e., high LCBD) communities.

Stability of macrofauna communities differed between taxa, but the differences depended on the region. Generally, stability was lower in the northern region, presumably reflecting the harsher environmental conditions (see above). Lumbricidae were an exception, with higher stability in the central region and similar stability in the other two regions, but their stability was not related to any of the potential drivers studied. Presumably, Lumbricidae are little affected by biotic interactions as they live in enemy‐free space due to their large body size and burrowing behavior. In addition, they do not have to compete for resources as they utilize organic matter that is available in excess, and they possess adaptive strategies to cope with detrimental abiotic conditions such as drought or cold (Holmstrup, 2003; Tilikj et al., 2024), thereby representing themselves biotic buffers against unstable ecosystem dynamics (Schwarzmüller et al., 2015). Despite mostly synchronous fluctuations and neutral variance ratios, the stability of Chilopoda was significantly related to species asynchrony, suggesting stabilizing dynamics in line with the portfolio effect.

Overall, soil meso‐ and macrofauna in forests did not follow a general declining trend of density and species richness; rather, they were influenced by regional climatic fluctuations, in particular precipitation (mesofauna) as well as regional soil conditions and forest type (both meso‐ and macrofauna). Notably, management intensity had only minor effects on the density and species richness of meso‐ and macrofauna, suggesting that changes in forest management may lead to changes in community composition, but not to a general decline in soil animal density and diversity (Junggebauer, Bluhm, et al., 2024). It has been shown before that land‐use exerts stronger effects on above‐ than belowground communities, and this applies in particular to forests (Brooks et al., 2012; Le Provost et al., 2021). This may be due to the relatively stable conditions in forest soils, with high abundance and diversity of resources, and comparatively minor changes of resources and abiotic conditions under different management intensities (Bardgett, 2002; Pollierer et al., 2021). The buffering effect of the soil habitat may explain why we did not observe a similar decline in diversity trends as those reported in many recent studies on above‐ground arthropods. On the other hand, the evidence of biodiversity decline in previous studies may be partly biased due to the underestimation of trend uncertainty and other methodological shortcomings, for example, the use of activity‐based rather than area‐based methods (Desquilbet et al., 2020; Johnson et al., 2024). Importantly, it should be noted that our study is confined to forests in Germany and therefore only represents the temperate, seasonal climate of continental Europe, whereas the broader topic of insect decline is based on global evidence. In addition, we only considered a restricted temporal window of 12 years, which may not be sufficient to capture long‐term declines. Therefore, our interpretations and conclusions must be viewed in light of these limitations, and future research should aim to compare soil fauna dynamics over longer time scales and across climatic zones such as boreal and subtropical regions. Nevertheless, as we showed that the effects of precipitation on soil mesofauna depended on forest management intensity, presumably due to differing susceptibility of corresponding leaf litter and soil habitats to drought, more pronounced shifts in management, that is, via clearcutting or conversion to agricultural fields/grassland, will likely increase the vulnerability of the soil fauna to adverse environmental conditions (Junggebauer, Gericke, et al., 2024). As the density and diversity of soil mesofauna responded sensitively to changes in precipitation regimes, soil mesofauna will likely be more affected by changes in long‐term precipitation regimes than macrofauna. Heavy rainfall events and longer dry periods due to climate change and associated shifts in microclimate due to tree dieback will likely detrimentally affect soil mesofauna diversity and shift community composition in the long term, as some species may not be able to recover after longer dry periods. Therefore, on a global scale, homogenization mechanisms and land‐use conversion are likely to negatively affect the overall abundance and diversity of soil animals. As a consequence, the altered trophic structure of decomposer communities may lead to shifts in important ecosystem functions such as decomposition. We demonstrated that detrimental impacts can be mitigated by higher species diversity, as the portfolio effect plays an important role in stabilizing meso‐ and macrofauna communities, even though populations usually fluctuate synchronously. This emphasizes the importance of maintaining and promoting the most diverse forest ecosystems possible, as these ecosystems also promote high biodiversity in the soil and are thus better able to ensure a variety of ecosystem functions.

## AUTHOR CONTRIBUTIONS

Stefan Scheu designed the study. Melanie M. Pollierer, André Junggebauer, Sarah Bluhm, Melissa Jüds, and Bernhard Klarner collected and identified soil animals and prepared the final data. Data were analyzed by Melanie M. Pollierer and André Junggebauer. Melanie M. Pollierer led the writing of the manuscript, and all authors contributed to drafts and approved the final version of the manuscript.

## CONFLICT OF INTEREST STATEMENT

The authors declare no conflicts of interest.

## Supporting information


Appendix S1.

## Data Availability

Data (Pollierer et al., 2025a) are available in Dryad at https://doi.org/10.5061/dryad.8w9ghx3w9. Code (Pollierer et al., 2025b) is available in Zenodo at https://doi.org/10.5281/zenodo.12796315.
