# Supplementary material for: Different patterns, but no temporal decline in temperate forest soil meso‐ and macrofauna over the last decade
Source: Ecology. 2025 Nov 12;106(11):e70246. doi: 10.1002/ecy.70246 (PMC12611797; doi:10.1002/ecy.70246)
Supplement: Supplementary file 1 — Appendix S1. [file ECY-106-e70246-s001.pdf]

Appendix S1: Supplementary Information

Different patterns, but no temporal decline in temperate forest soil meso- and macrofauna over the last decade

Melanie M. Pollierer, André Junggebauer, Sarah Bluhm, Melissa Jüds, Bernhard Klarner, Stefan Scheu

Ecology

Contents **Page**

**Supplementary Section S1** – Study sites and environmental variables	**2**
**Supplementary Section S2** – Local contribution to beta diversity	**4**
**Supplementary Figure S1** – Relationship between mean LCBD and stability	**5**
**Supplementary Figure S2** – The Silvicultural Management Intensity indicator (SMI)	**6**
**Supplementary Figure S3** – Estimated sampling completeness of mesofauna	**7**
**Supplementary Figure S4** – Estimated sampling completeness of macrofauna	**8**
**Supplementary Figure S5** – Mean-variance scaling relationships of mesofauna taxa	**9**
**Supplementary Figure S6** – Mean-variance scaling relationships of macrofauna taxa	**10**
**Supplementary Figure S7** – Principal component analysis of environmental variables	**11**
**Supplementary Figure S8** – Mesofauna density and richness as influenced by precipitation	**12**
**Supplementary Figure S9** – Mesofauna density as influenced by the SMI	**13**
**Supplementary Figure S10** – Macrofauna density and gamma diversity in different years	**14**
**Supplementary Figure S11** – Macrofauna density in different years and regions	**15**
**Supplementary Figure S12** – Temporal trends of macrofauna density as influenced by SMI	**16**
**Supplementary Figure S13** – Macrofauna richness in different taxa and regions	**17**
**Supplementary Figure S14** – Effect of SMI on macrofauna richness in different regions	**18**
**Supplementary Figure S15** – Mesofauna stability as influenced by winter precipitation in different regions	**19**
**Supplementary Table S1** – Results of linear mixed-effects model of mean LCBD on mesofauna stability	**20**
**Supplementary Table S2** – Observed sample coverage of mesofauna taxa and macrofauna	**21**
**Supplementary Table S3** – Results of generalized linear mixed models on mesofauna density and richness	**22**
**Supplementary Table S4** – Estimated effects of precipitation on mesofauna density and richness	**23**
**Supplementary Table S5** – Results of generalized linear mixed models on macrofauna density and richness	**24**
**Supplementary Table S6** – Estimated effects of year on macrofauna density	**25**
**Supplementary Table S7** – Estimated effects of SMI on macrofauna density	**26**
**Supplementary Table S8** – Estimated effects of SMI on macrofauna richness	**27**
**Supplementary Table S9** – Results of linear models on meso- and macrofauna stability	**28**
**Supplementary Table S10** – Results of linear models on drivers of stability in mesofauna taxa	**29**
**Supplementary Table S11** – Results of linear models on drivers of stability in macrofauna taxa	**30**
**Supplementary Table S12** – Estimated effects of winter precipitation on mesofauna stability	**31**
**Supplementary References**	**32**

Section S1: *Study sites and environmental variables*

The Schorfheide is a young glacial landscape with an altitude of 3–140 m a.s.l., a mean annual temperature of 8.0–8.5°C and a mean annual precipitation of 500–600 mm. The climate is subcontinental with hot and dry summers and cold winters (Natkhin et al., 2012). Soils in the Schorfheide are mainly cambisols. The Hainich is based on calcareous bedrock, with an altitude of 285 to 550 m a.s.l. Soils in the Hainich are mainly luvisols with few stagnosols. The mean annual temperature is 6.5–8.0°C and the mean annual precipitation is 600–800 mm. The Swabian Alb varies 460 to 860 m a.s.l. in altitude, has a mean annual temperature of 6–7°C and a mean annual precipitation of 700–1,000 mm. It is also based on calcareous bedrock, but with karst phenomena. Its soils are cambisols and leptosols. Acidity of the soil ranged from pH 3.3 ± 0.19 in the Schorfheide to 4.51 ± 0.72 in the Swabian Alb to 4.59 ± 0.67 in the Hainich. In each region, 16 forest plots (48 plots total) were sampled, covering a gradient of management intensity which was quantified by the silvicultural management intensity indicator (SMI, taken from the Biodiversity Exploratories database (BExIS)) that includes a risk and density component, and accounts for tree species, tree age and aboveground living biomass (Schall & Ammer, 2013, 2023; Appendix S1: Fig. S1). Forests included unmanaged beech forests (age ~120 years, unmanaged for at least 60 years), managed age class beech forests (*Fagus sylvatica*, ages from ~30 to 80 years) and coniferous forests consisting of spruce (*Picea abies*; age ~60 years) in the southern and central region, and pine (*Pinus sylvestris*; age ~50 years) in the northern region. The study sites were located within 100 m × 100 m grid plots established as core sampling sites of the Biodiversity Exploratories. The plots were at least 200 m apart from each other and had a minimum distance of 100 m to the next forest edge. Precipitation for each year and sampling site was derived from the RADOLAN (Radar Online Adjustment) product of the German Weather Service (Deutscher Wetterdienst), which provides hourly radar-based precipitation estimates for Germany adjusted to rain gauge data on a 1 km² grid (Kreklow et al., 2019). Temperature

was measured with environmental sensors installed at 2 m above the ground at all sites (Fischer et al., 2010). We calculated mean values for temperature and precipitation in winter (December to February) and spring (March to May). Microbial biomass (C_{mic}) in leaf litter and soil was assessed by measuring the maximum initial respiratory response (MIRR; mg O_2 g^{-1} h^{-1}) after glucose addition (SIR method; Anderson & Domsch, 1978) in an automated O_2 micro-compensation apparatus (Scheu, 1992). Glucose (80 and 10 mg g^{-1} dry weight for litter and soil, respectively) was added as an aqueous solution to approximately 1 g of leaf litter material (Beck et al., 1997).

Section S2: *Local contribution to beta diversity*

To obtain a summary measure of compositional uniqueness, we calculated the mean Local Contribution to Beta Diversity (mean LCBD) for each plot by averaging LCBD values across all sampling years. This plot-level metric reflects how consistently unique the community composition was over time. To explore the relationship between plot-level compositional uniqueness and community temporal stability across taxa, we conducted a linear mixed-effects model including mean LCBD, taxon, and Region as fixed effects, and plot as a random effect. The model revealed a significant main effect of mean LCBD on stability ($F_{1,114}$ = 19.70, $p < 0.001$), suggesting that plots with more consistently unique community composition over time tended to exhibit lower stability. This effect varied significantly across taxa (mean LCBD × taxon interaction: $F_{2,116}$ = 3.27, $p = 0.042$), with post hoc inspection indicating that the negative relationship was most pronounced in Mesostigmata (Fig. S1). Due to collinearity between mean LCBD and effective species diversity (inverse Simpson), and because LCBD is derived from relative abundance matrices, we excluded LCBD from the main models to avoid confounding. These findings are instead provided as a complementary analysis.

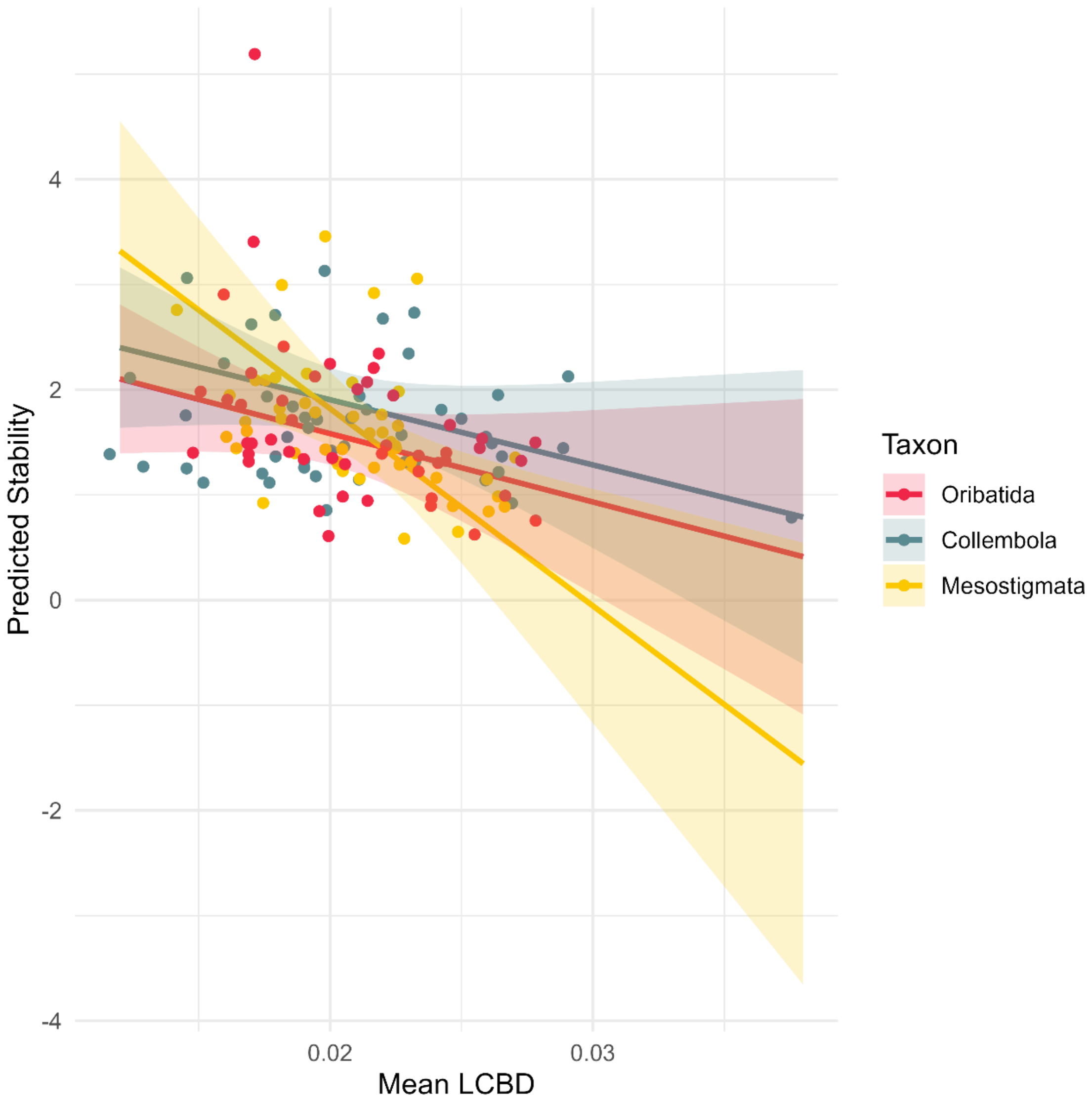

Fig. S1: Predicted linear relationship between mean LCBD and stability across mesofauna taxa from the linear mixed-effects model (Table S1). Lines represent model-predicted values for each taxon, with shaded areas showing 95% confidence intervals, points represent observed values. Predictions were made holding other variables constant and marginalizing over random effects.

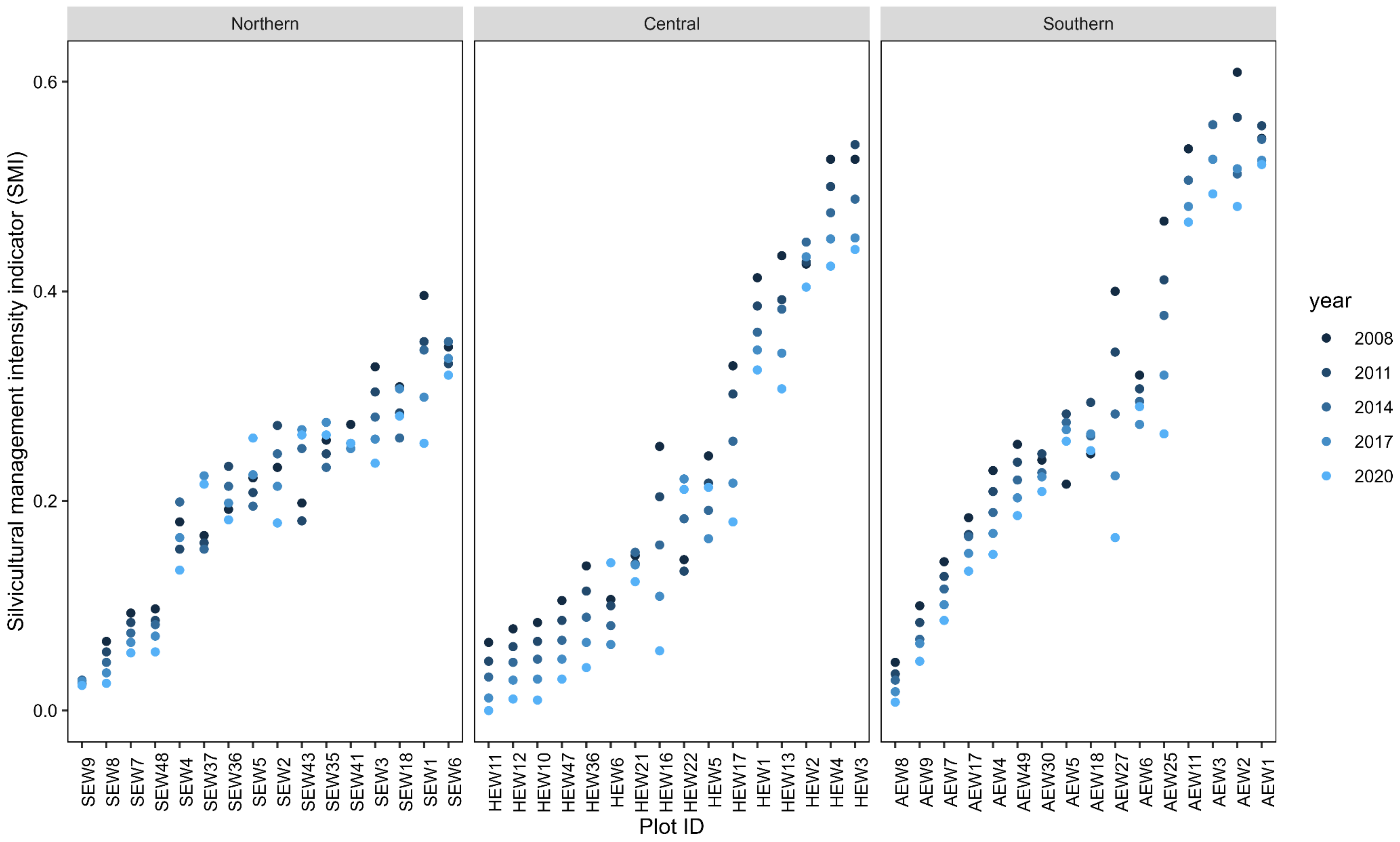

Fig. S2: The silvicultural management intensity indicator (SMI) of all sampling plots in the five sampling years, separated by regions (northern, central and southern Germany).

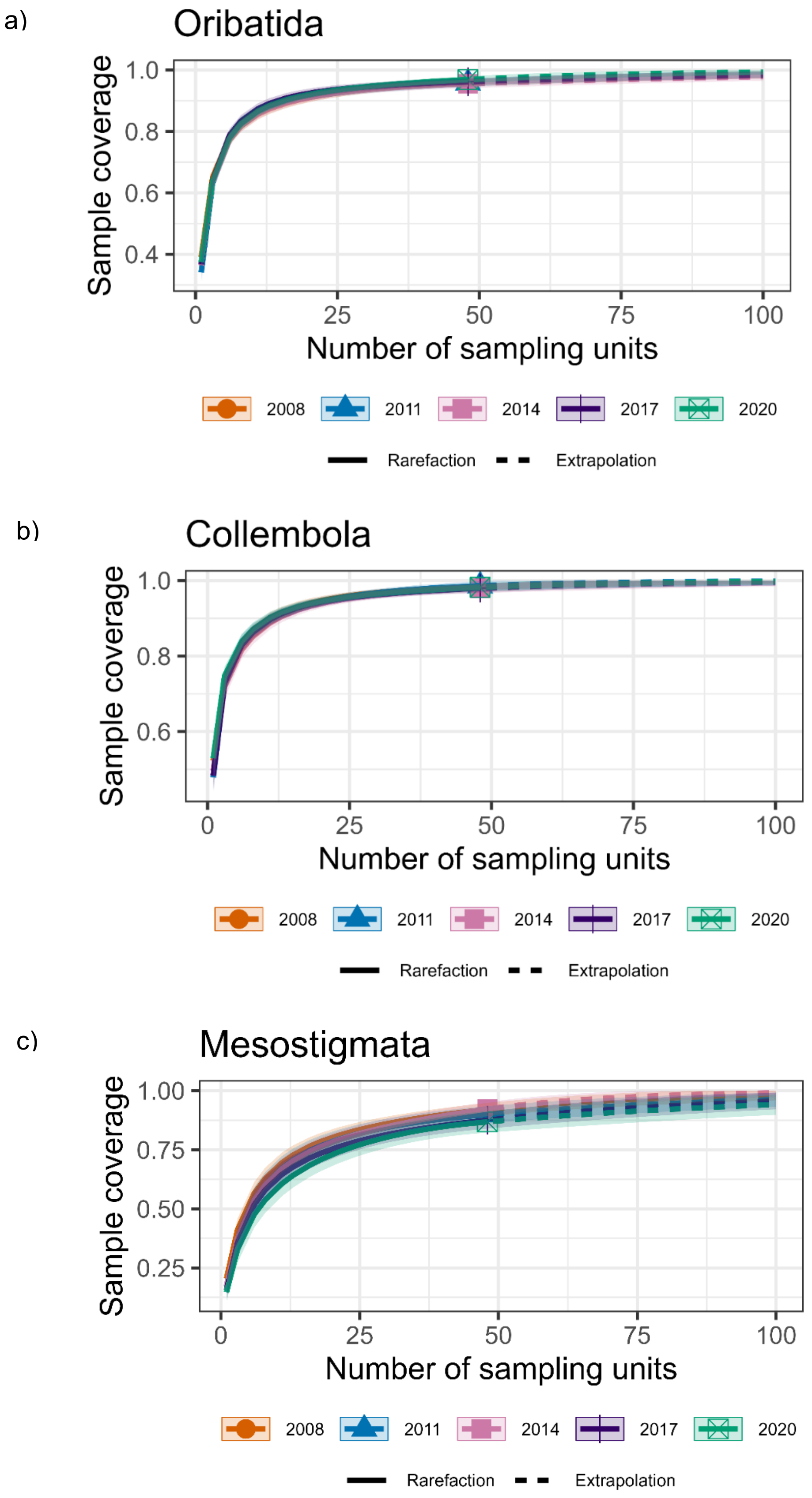

Fig. S3: Estimated sample completeness curves for the mesofauna taxa (a) Oribatida, (b) Collembola and (c) Mesostigmata in each of the five sampling years. Estimations are based on incidence data from the 48 soil cores taken each year and extrapolated to the double of the reference sample size.

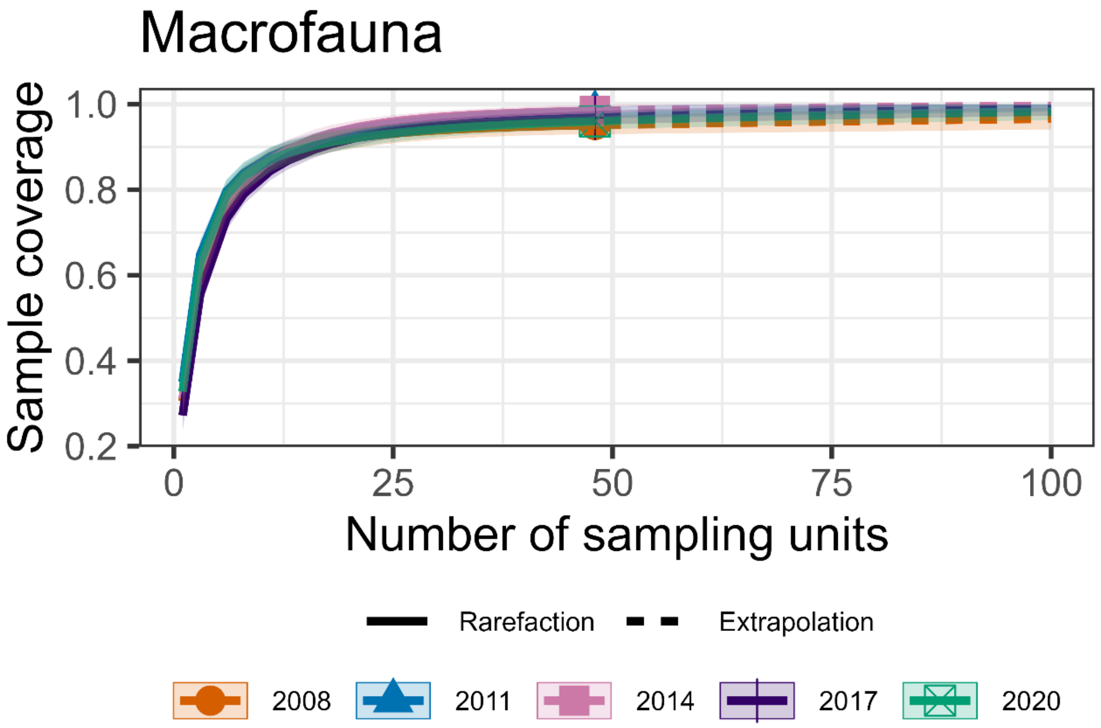

Fig. S4: Estimated sample completeness curves for macrofauna in each of the five sampling years. Estimations are based on incidence data from the 48 soil cores taken each year and extrapolated to the double of the reference sample size.

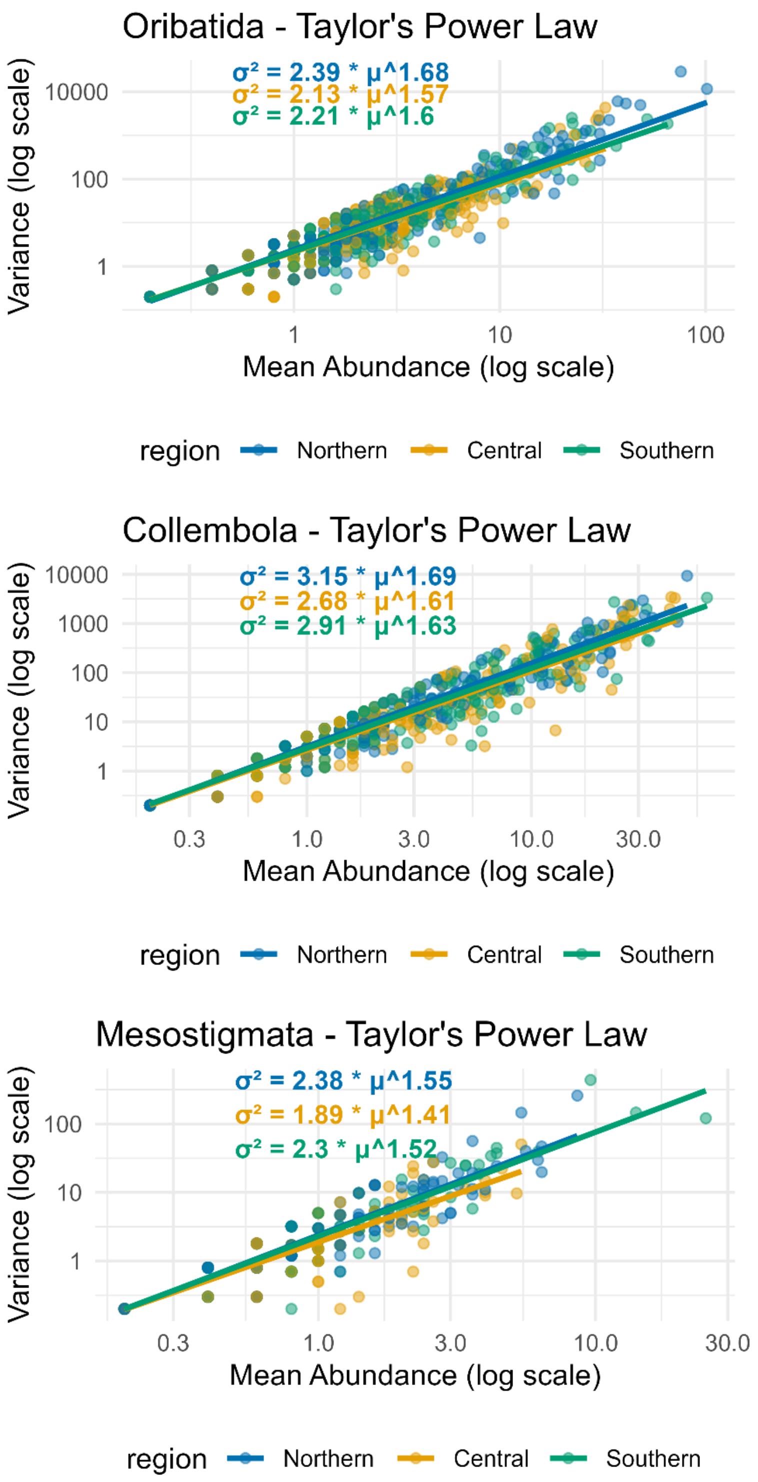

Fig. S5: Mean-variance scaling relationships of mesofauna taxa (Oribatida, Collembola, Mesostigmata) in northern, central and southern regions of Germany, with each dot representing the mean abundance and variance of a species within a plot. The exponent of μ in the equation of Taylor's power law represents the z-value.

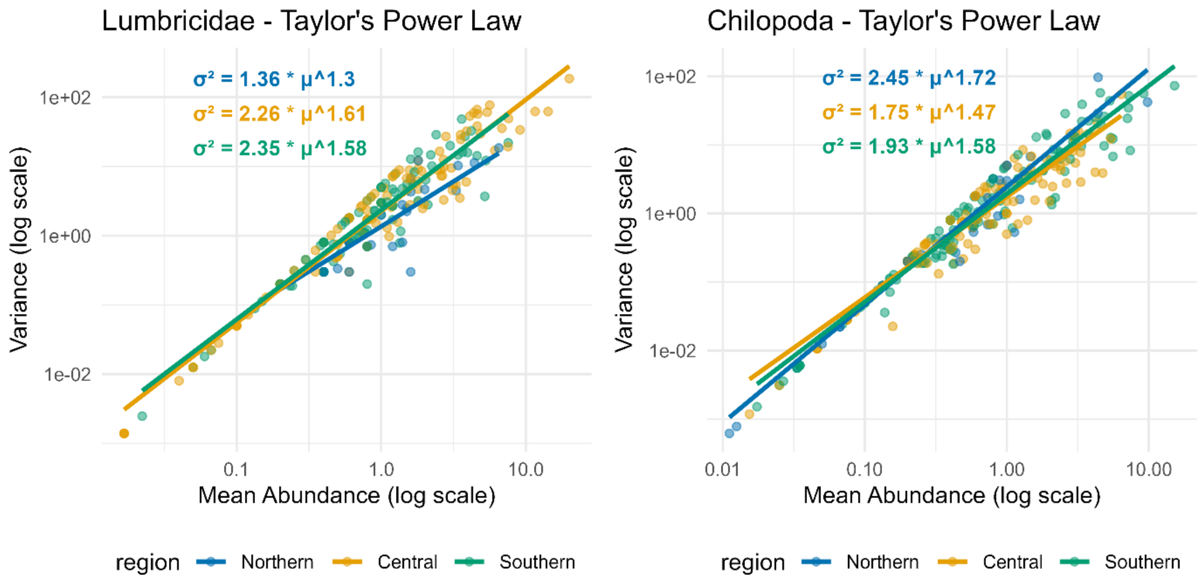

Fig. S6: Mean-variance scaling relationships of macrofauna taxa (Lumbricidae, Chilopoda) in northern, central and southern regions of Germany, with each dot representing the mean abundance and variance of a species within a plot. The exponent of mu in the equation of Taylor’s Power Law represents the z-value.

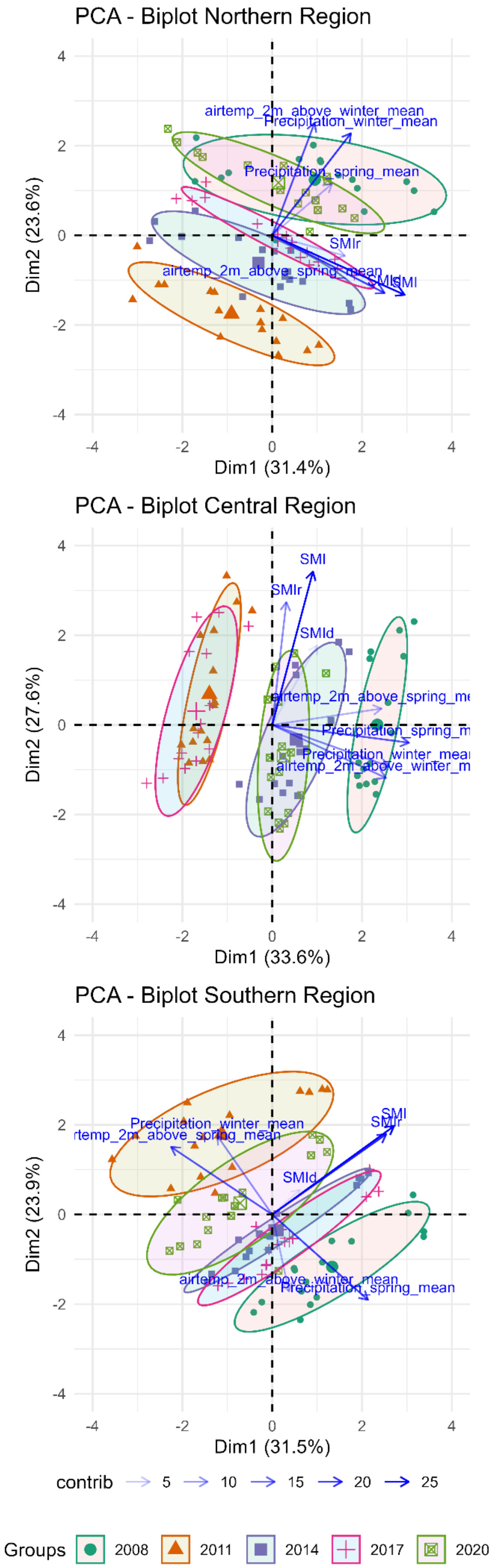

Fig. S7: Principal component analyses of environmental variables (mean air temperature 2 m above the soil and mean precipitation in winter and spring, respectively, and the silvicultural management intensity indicator (SMI) with its risk (SMI_r) and density (SMI_d) components of each of 16 plots in the northern (a) , central (b) and southern (c) region of Germany. Environmental variables were measured in three-year intervals from 2008 to 2020. The relative strength of contribution is indicated by the length and transparency of the arrows.

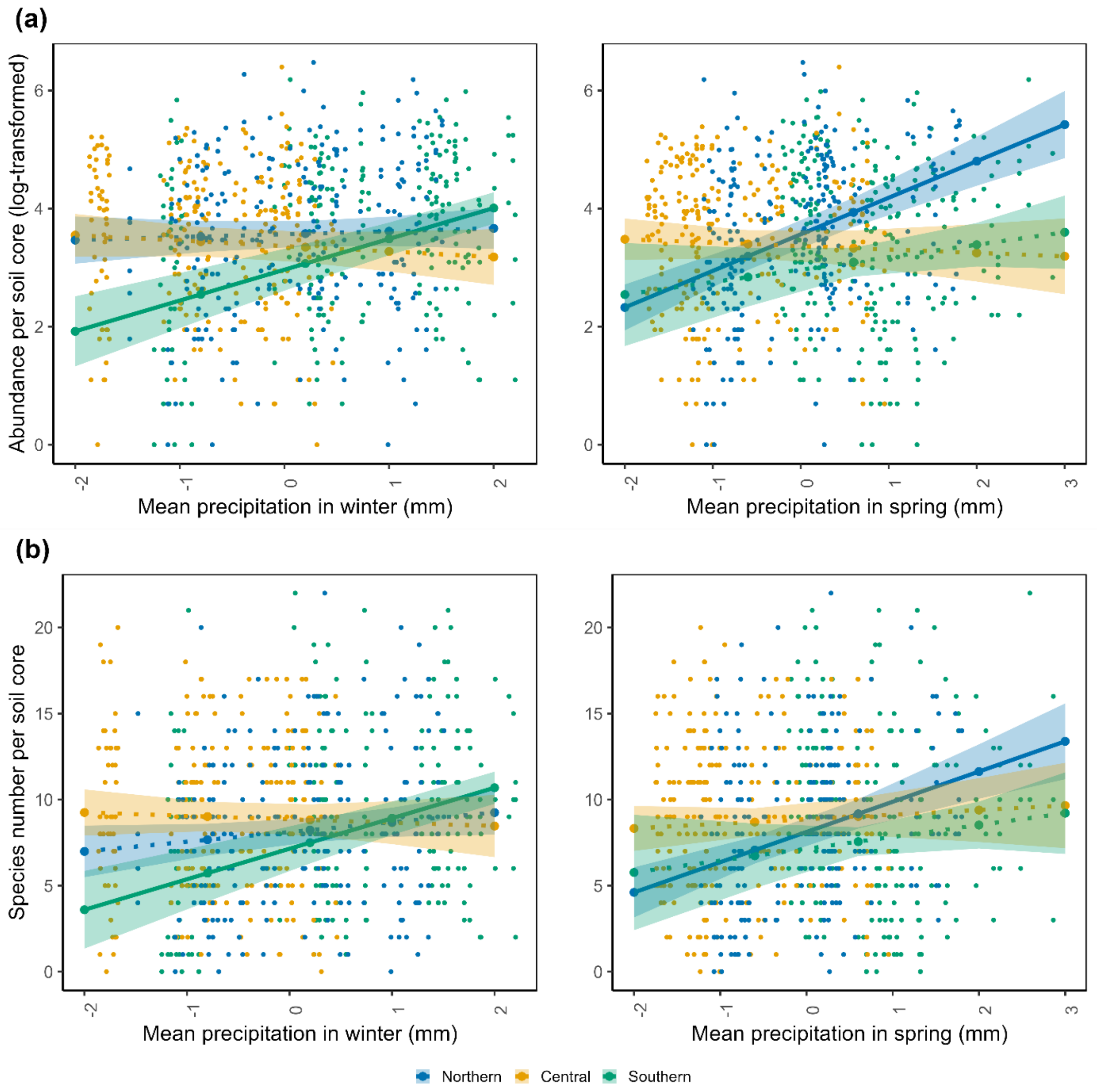

Fig. S8: Abundance (a) and species richness (b) of soil mesofauna taxa in northern, central and southern regions in Germany as influenced by mean precipitation of the preceding winter months and mean precipitation in spring, i.e. the time of sampling. Points represent observed values; lines show model-predicted trends from generalized linear mixed-effects models, and shaded areas indicate 95% confidence intervals. Solid lines indicate significant effects (see Table S4 for estimates, standard errors, test statistics, degrees of freedom, and p-values).

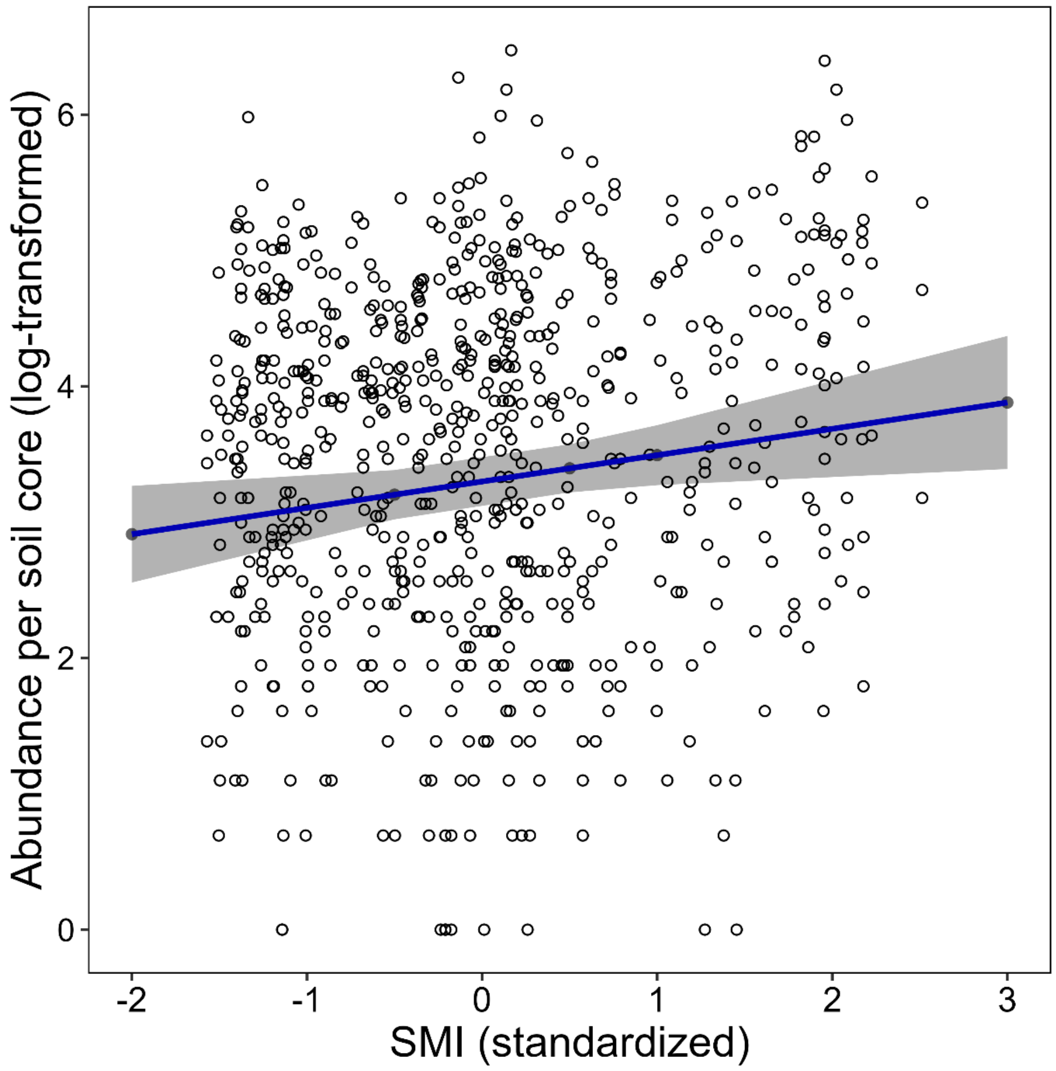

Fig. S9: The effect of the silvicultural management intensity index (SMI) on density (abundance per soil core, log-transformed) of soil mesofauna as estimated by the full linear mixed-effects model (Table S3). Model predictions with 95% confidence intervals and raw data points are shown.

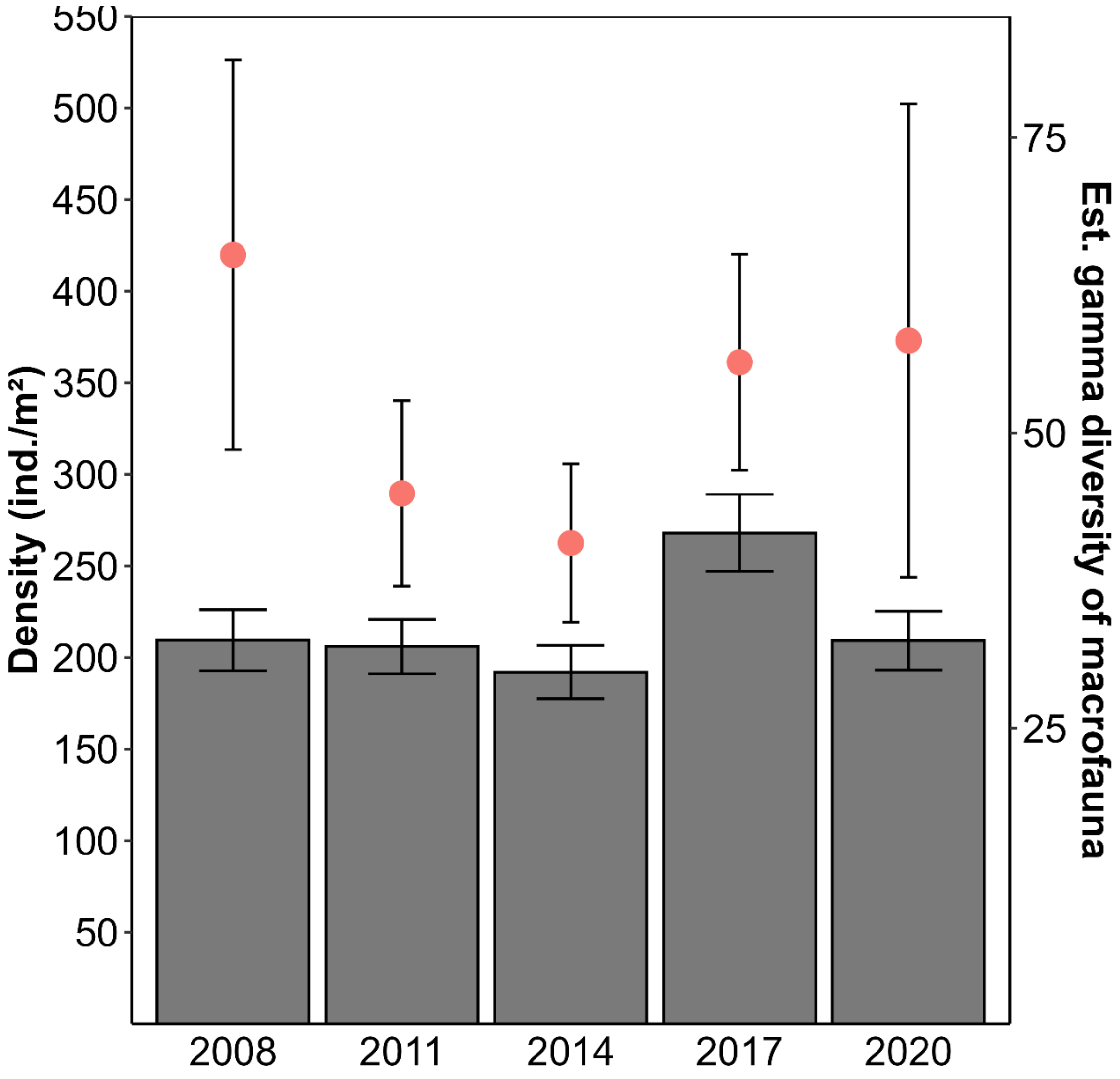

Fig. S10: Mean density (bars, ±SE) and estimated gamma diversity (orange dots with 95 % confidence intervals) of soil macrofauna sampled in three-year intervals from 2008 to 2020.

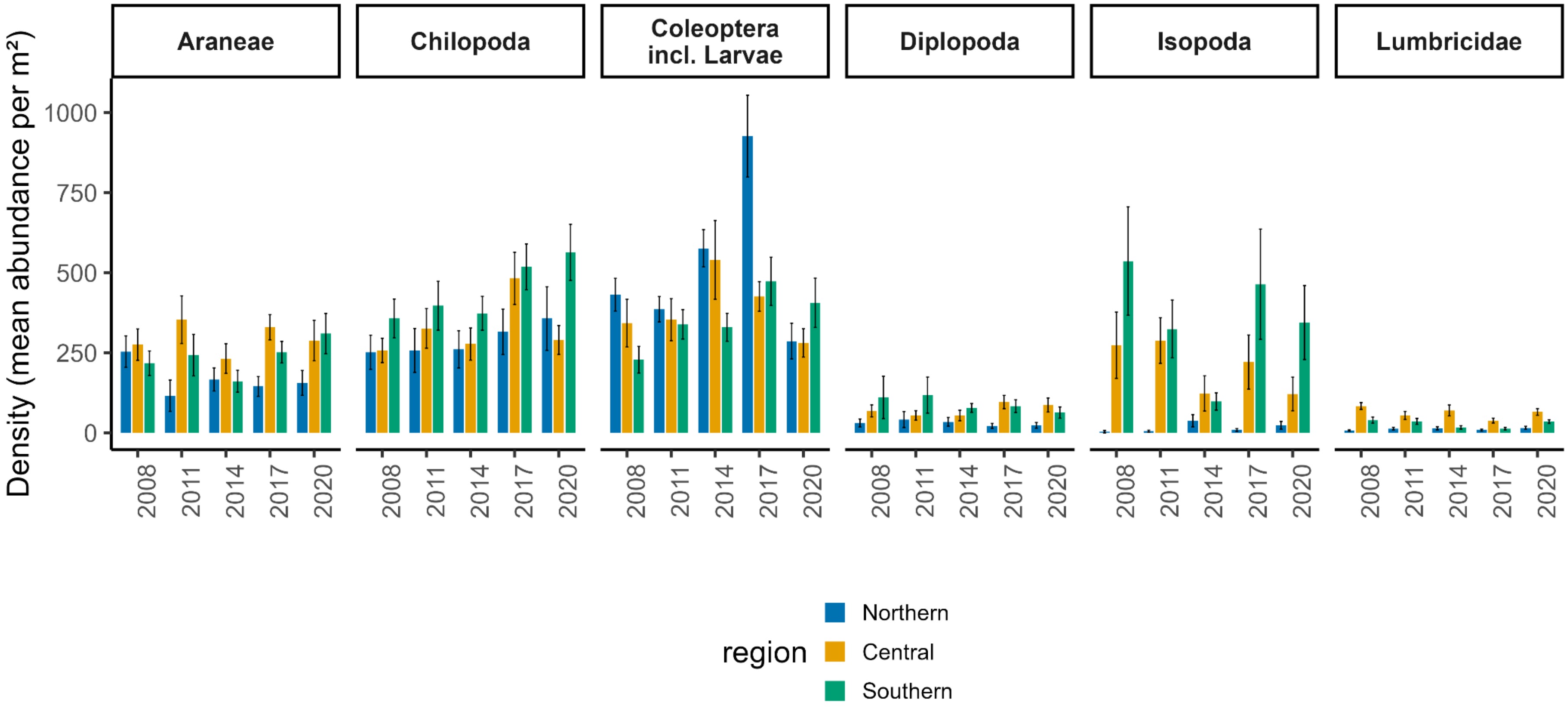

Fig. S11: Mean density of soil macrofauna omnivores/predators (Coleoptera, Chilopoda, Araneae) and decomposers (Isopoda, Diplopoda, Lumbricidae) in the studied northern, central and southern region of Germany in different years.

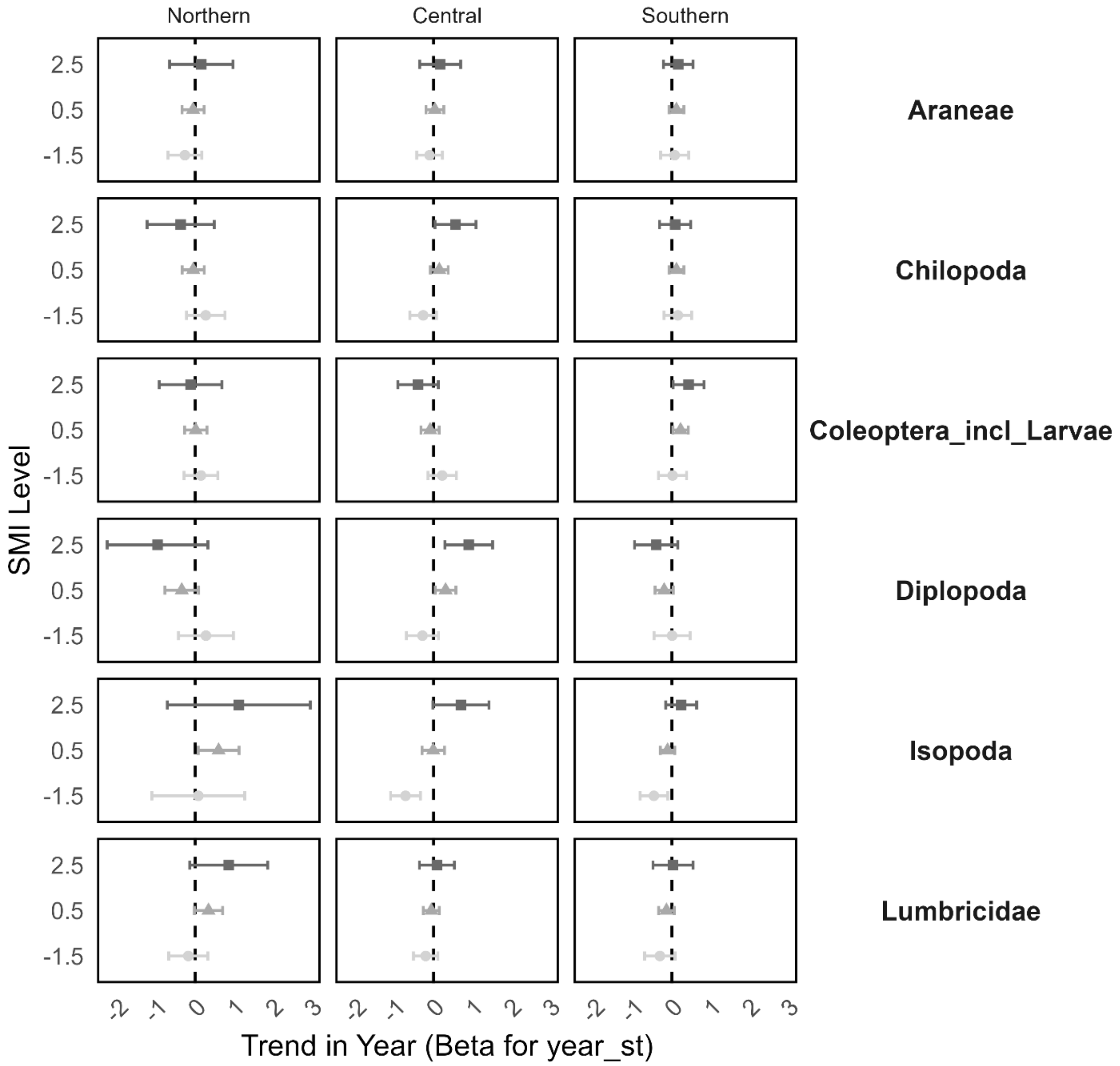

Fig. S12: Temporal trends (effect of year) of macrofauna density at different levels of forest management intensity (SMI) on density for different macrofauna taxa across regions. The plot shows the estimated effect sizes (β values) of SMI (Sylvicultural Management Index) on animal density, derived from a generalized linear mixed model. Each point represents the effect size (β) for a specific taxon in a given region, with the x-axis displaying the region and the y-axis representing the magnitude and direction of the effect (β for SMI_{st}). Error bars indicate 95% confidence intervals for the estimated effect sizes. Positive β values indicate an increase in density with higher SMI, while negative β values indicate a decrease.

a)

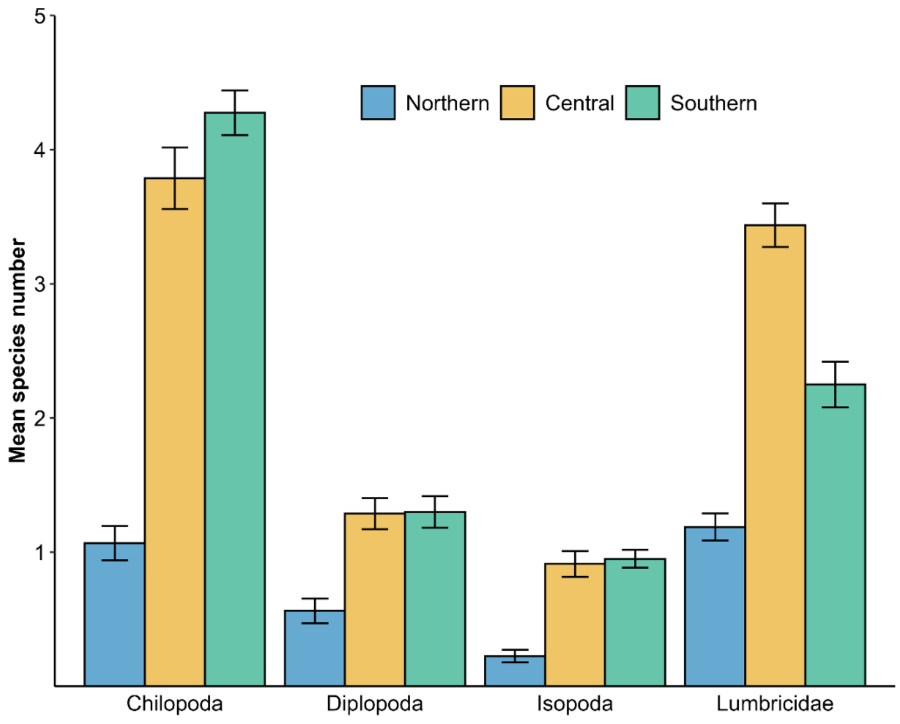

b)

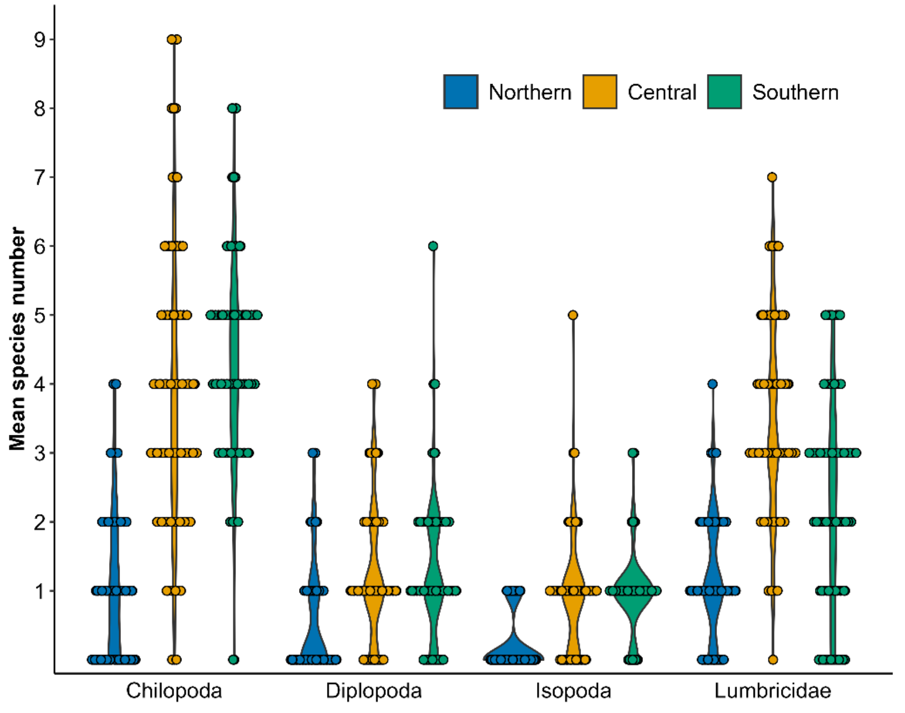

Fig. S13: a) Mean (± SE) species richness (species number per soil core/per 0.25 m² for Lumbricidae) and b) the distribution of species richness across taxa of soil macrofauna predators (Chilopoda) and decomposers (Diplopoda, Isopoda, Lumbricidae) in the studied northern, central and southern region of Germany, In b), the width of each violin represents the density of data points. Individual data points are overlaid using a quasirandom jitter to display the raw observations..

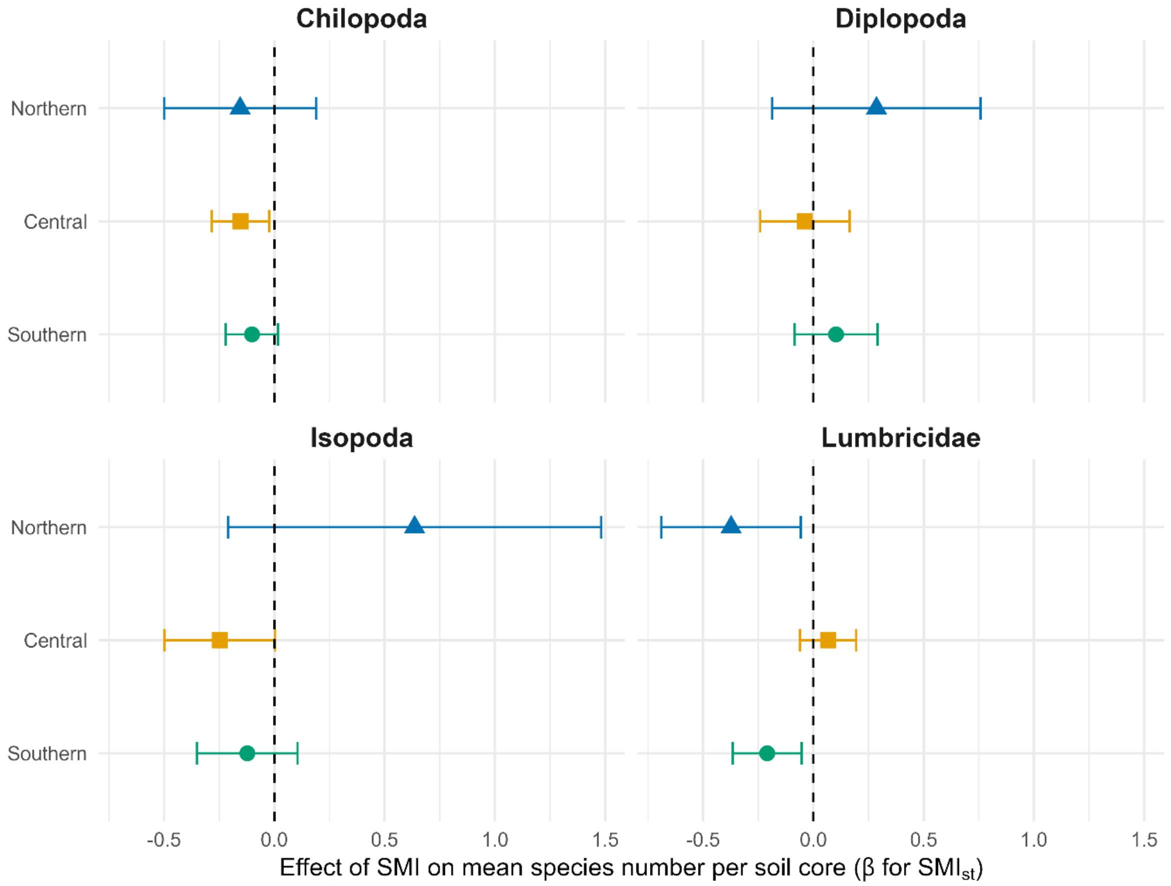

Fig. S14: Effect of silvicultural management intensity (SMI) on mean species number per soil core of macrofauna taxa in the studied northern, central, and southern regions of Germany. Estimated effect sizes (β values) of standardized SMI are derived from a generalized linear mixed-effects model. Points and error bars represent the estimated model coefficients (β) and 95% confidence intervals, respectively, for each taxon in a given region. Positive β values indicate increasing species richness with increasing SMI, while negative β values indicate decreasing species richness. Effect estimates were obtained using the emtrends() function from the emmeans package. Statistical details for each estimate (standard error, z-ratio, p-value) are provided in Table S8.

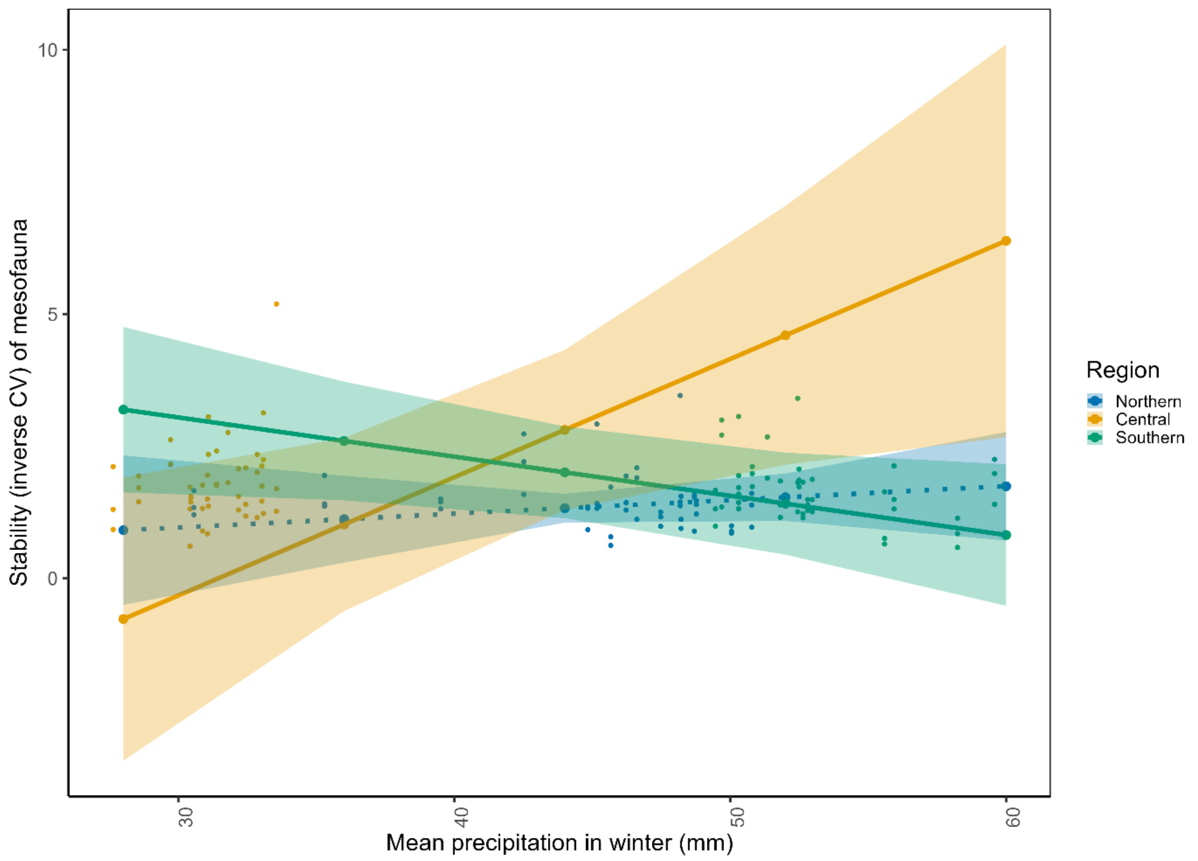

Fig. S15: Stability (inverse coefficient of variation) of mesofauna in the studied northern, central and southern region of Germany as influenced by the mean precipitation in winter (mm). Points represent observed values; lines show model-predicted trends from a generalized linear mixed-effects model (Table S9), and shaded areas indicate 95% confidence intervals. Solid lines indicate significant effects (see Table S12 for estimates, standard errors, test statistics, degrees of freedom, and p-values).

Table S1: Type III Analysis of Variance (ANOVA) results from a linear mixed-effects model examining the effects of mean LCBD (Local Contribution to Beta Diversity), taxon, region, and their interactions on stability (indicated by the inverse coefficient of variation) of mesofauna taxa. The table shows sum of squares, numerator and denominator degrees of freedom (num df, den df), F-values, and p-values calculated using Satterthwaite's method. Significant effects are indicated in bold.

Factor	Sum of squares	num df	den df	F-value	p-value
**Mean LCBD**	6.42	1	114.08	19.70	**<0.001**
Taxon	1.98	2	115.80	3.04	0.052
Region	1.11	2	111.79	1.70	0.187
**Mean LCBD × Taxon**	2.13	2	116.19	3.27	**0.042**
Mean LCBD × Region	0.96	2	113.64	1.48	0.232
Taxon x Region	1.69	4	115.86	1.30	0.276
Mean LCBD × Taxon × Region	1.34	4	116.10	1.03	0.395

Table S2: Observed sample coverage (n = 48) of mesofauna taxa (Oribatida, Collembola, Mesostigmata) and macrofauna sampled in three-year intervals from 2008 to 2020.

	**Oribatida**	**Collembola**	**Mesostigmata**	**Macrofauna**
Year	Sample coverage (%)	Sample coverage (%)	Sample coverage (%)	Sample coverage (%)
2008	95.8	98.4	92.0	95.5
2011	95.8	98.6	89.9	98.1
2014	95.5	97.9	92.2	98.4
2017	96.2	98.0	87.5	97.1
2020	96.9	98.2	86.8	95.7

Table S3: Results of linear mixed-effects models on the response of mesofauna density and species richness to the factors year of sampling (Year), taxon (Oribatida, Collembola, Mesostigmata), region (northern, central, southern), forest management intensity (Sylvicultural Management Index – SMI), precipitation in the previous winter (precipitation winter) or at the time of sampling (precipitation spring) and microbial biomass of leaf litter (C_{mic} leaf litter). Factors that significantly affected either density or richness and significant p-values are shown in bold.

Factor	**Mesofauna density**					**Mesofauna richness**				
	Sum of squares	num df	den df	F-value	p-value	Sum of squares	num df	den df	F-value	p-value
Year	0.97	1	663.95	1.63	0.202	7.50	1	666.53	0.80	0.371
**Taxon**	534.14	2	622.86	448.03	**<0.001**	5990.30	2	627.04	320.25	**<0.001**
**Region**	4.82	2	87.81	4.04	**0.021**	45.96	2	105.21	2.46	0.091
**SMI**	3.55	1	108.24	5.95	**0.016**	4.86	1	117.12	0.52	0.472
**Precipitation winter**	7.28	1	650.15	12.21	**0.001**	146.78	1	660.98	15.69	**<0.001**
**Precipitation spring**	9.67	1	662.97	16.22	**<0.001**	120.98	1	668.87	12.94	**<0.001**
Cmic leaf litter	0.26	1	647.99	0.43	0.513	0.04	1	597.27	0.00	0.945
Year × Taxon	2.76	2	622.86	2.32	0.099	26.65	2	627.04	1.43	0.241
**Year × Region**	12.44	2	660.46	10.43	**<0.001**	74.11	2	661.24	3.96	**0.019**
Year × SMI	0.10	1	647.57	0.17	0.676	0.17	1	658.50	0.02	0.894
Taxon × Region	5.10	4	622.86	2.14	0.075	43.30	4	627.04	1.16	0.328
Taxon × SMI	2.47	2	622.86	2.07	0.127	6.32	2	627.04	0.34	0.714
Taxon × Cmic leaf litter	1.35	2	622.86	1.13	0.324	2.15	2	627.04	0.12	0.891
Region × SMI	0.51	2	104.41	0.43	0.651	1.37	2	109.03	0.07	0.930
**Region × Precipitation winter**	20.16	2	652.64	16.91	**<0.001**	189.75	2	662.74	10.14	**<0.001**
**Region × Precipitation spring**	19.57	2	646.32	16.42	**<0.001**	96.73	2	647.67	5.17	**0.006**
SMI × Precipitation winter	0.13	1	663.66	0.21	0.645	10.66	1	668.76	1.14	0.286
**SMI × Precipitation spring**	2.87	1	650.84	4.81	**0.029**	30.92	1	661.95	3.31	0.069
Year × Taxon × Region	2.61	4	622.86	1.10	0.357	56.86	4	627.04	1.52	0.195
Year × Taxon × SMI	0.19	2	622.86	0.16	0.853	19.36	2	627.04	1.04	0.356
Year × Region × SMI	2.16	2	646.86	1.81	0.164	15.44	2	656.73	0.83	0.438
**Taxon × Region × SMI**	3.38	4	622.86	1.42	0.226	103.57	4	627.04	2.77	**0.027**
**Region × SMI × Precipitation winter**	4.45	2	657.03	3.74	**0.024**	35.01	2	662.69	1.87	0.155
Region × SMI × Precipitation spring	1.86	2	651.09	1.56	0.211	15.86	2	660.07	0.85	0.429
Year × Taxa × Region × SMI	1.10	4	622.86	0.46	0.764	10.56	4	627.04	0.28	0.889

Table S4: Estimated effects of mean precipitation of the preceding winter months and mean precipitation in spring, i.e. the time of sampling, on mesofauna density and species richness per region. Estimated trends and 95% confidence intervals (CIs) were extracted using the emtrends() function from GLMMs with a negative binomial distribution. Results are shown for each region (northern, central, southern), separately for mesofauna density (abundance per soil core) and species richness (species number per soil core), in response to mean winter and spring precipitation. Significant effects ($p < 0.05$) are indicated in bold. For full model results, see Table S2. For plots of estimated effects and raw data see Fig. S8.

**Response**	**Precipitation**	**Region**	Trend Estimate	SE	df	95% CI (lower–upper)	p-value
Density	Winter	Northern	0.050	0.078	656.0	-0.103 – 0.203	0.523
Density	Winter	Central	-0.092	0.086	628.9	-0.262 – 0.077	0.2840
Density	Winter	Southern	0.522	0.075	664.7	0.374 – 0.669	**<0.0001**
Density	Spring	Northern	0.619	0.086	640.0	0.450 – 0.789	**<0.0001**
Density	Spring	Central	-0.058	0.087	638.0	-0.229 – 0.114	0.509
Density	Spring	Southern	0.211	0.145	665.8	-0.074 – 0.497	0.147
Richness	Winter	Northern	0.564	0.306	665.0	-0.037 – 1.166	0.066
Richness	Winter	Central	-0.197	0.342	635.0	-0.867 – 0.474	0.441
Richness	Winter	Southern	1.773	0.294	667.0	1.197 – 2.350	**<0.001**
Richness	Spring	Northern	1.756	0.342	647.4	1.085 – 2.428	**<0.0001**
Richness	Spring	Central	0.266	0.345	645.6	-0.411 – 0.943	0.441
Richness	Spring	Southern	0.690	0.565	656.1	-0.421 – 1.800	0.223

Table S5: Results of generalized linear mixed models on the response of macrofauna density and species richness to the factors year of sampling (Year), taxon (Isopoda, Diplopoda, Lumbricidae, Chilopoda, Araneae, Coleoptera), region (northern, central, southern), forest management intensity (Sylvicultural Management Index – SMI) and microbial biomass of leaf litter (C_{mic} leaf litter).

	**Macrofauna density**			**Macrofauna richness**		
Factor	Chi-square	Df	p-value	Chi-square	Df	p-value
**(Intercept)**	401.93	1	**<0.001**	107.71	1	**<0.001**
Year	3.84	1	0.050	1.10	1	0.294
**Taxon**	131.42	5	**<0.001**	202.23	3	**<0.001**
**Region**	169.77	2	**<0.001**	70.22	2	**<0.001**
**SMI**	1.14	1	0.285	6.91	1	**0.009**
Cmic leaf litter	0.02	1	0.884	0.10	1	0.746
**Year × Taxon**	14.39	5	**0.013**	2.38	3	0.497
**Year × Region**	9.51	2	**0.009**	1.37	2	0.504
**Year × SMI**	4.41	1	**0.036**	0.78	1	0.378
**Taxon × Region**	284.10	10	**<0.001**	28.98	6	**<0.001**
**Taxon × SMI**	24.65	5	**<0.001**	7.08	3	0.069
Taxon × Cmic leaf litter	5.72	5	0.334	3.42	3	0.331
**Region × SMI**	18.72	2	**<0.001**	11.19	2	**0.004**
**Year × Taxon × Region**	25.12	10	**0.005**	5.47	6	0.485
Year × Taxon × SMI	5.32	5	0.379	1.12	3	0.772
Year × Region × SMI	1.42	2	0.492	0.56	2	0.755
**Taxon × Region × SMI**	52.42	10	**<0.001**	19.10	6	**0.004**
**Year × Taxon × Region × SMI**	18.48	10	**0.047**	7.48	6	0.279

Table S6: Effects of year (standardized) on macrofauna density by taxon and region, based on generalized linear mixed-effects models (GLMMs) with a negative binomial distribution. The table presents estimated effects (β values) of year on macrofauna density per soil core, along with standard errors (SE), Wald z-ratios, asymptotic degrees of freedom (df), and p-values. Effects were extracted using the emtrends() function from the emmeans package, accounting for model interactions. Degrees of freedom are asymptotically infinite (∞) due to the use of Wald z-tests. Full model results are provided in Table S3. Estimated trends are visualized in Figure 3a of the main manuscript

Region	Taxon	Effect of year (standardized)	SE	z-ratio	Df	p-value
**Northern**	Araneae	-0.1049	0.1076	-0.975	∞	0.330
	Chilopoda	0.0220	0.1081	0.204	∞	0.838
	Coleoptera_incl_Larvae	0.0437	0.1118	0.391	∞	0.696
	Diplopoda	-0.2042	0.1613	-1.266	∞	0.206
	Isopoda	0.4814	0.2073	2.322	∞	**0.020**
	Lumbricidae	0.2211	0.1370	1.614	∞	0.107
**Central**	Araneae	0.0025	0.1029	0.024	∞	0.981
	Chilopoda	0.0529	0.1047	0.506	∞	0.613
	Coleoptera_incl_Larvae	-0.0161	0.1111	-0.145	∞	0.885
	Diplopoda	0.1728	0.1236	1.397	∞	0.162
	Isopoda	-0.1733	0.1184	-1.464	∞	0.143
	Lumbricidae	-0.0910	0.0972	-0.936	∞	0.349
**Southern**	Araneae	0.1088	0.1020	1.066	∞	0.286
	Chilopoda	0.1286	0.0994	1.294	∞	0.196
	Coleoptera_incl_Larvae	0.1781	0.1044	1.705	∞	0.088
	Diplopoda	-0.1502	0.1204	-1.247	∞	0.212
	Isopoda	-0.1914	0.0977	-1.960	∞	**0.050**
	Lumbricidae	-0.1771	0.0984	-1.799	∞	0.072

Table S7: Effects of silvicultural management intensity (standardized SMI) on macrofauna density by taxon and region, based on generalized linear mixed-effects models (GLMMs) with a negative binomial distribution. The table reports estimated effects (β values) of SMI on macrofauna density per soil core, along with standard errors (SE), Wald z-ratios, asymptotic degrees of freedom (df), and p-values. Effects were extracted using the emtrends() function from the emmeans package, accounting for model interactions. Degrees of freedom are asymptotically infinite (∞) due to the use of Wald z-tests. Full model results are provided in Table S3. Estimated trends are visualized in Figure 3b of the main manuscript.

Region	Taxon	Effect of SMI (standardized)	SE	z-ratio	Df	p-value
Northern	Araneae	0.4207	0.1827	2.302	∞	**0.021**
	Chilopoda	0.2096	0.1795	1.168	∞	0.243
	Coleoptera_incl_Larvae	-0.1738	0.1608	-1.081	∞	0.280
	Diplopoda	0.4202	0.2599	1.617	∞	0.106
	Isopoda	1.0404	0.3738	2.784	∞	**0.005**
	Lumbricidae	-0.4295	0.1858	-2.311	∞	**0.021**
Central	Araneae	-0.0997	0.107	-0.931	∞	0.352
	Chilopoda	-0.0538	0.1081	-0.498	∞	0.619
	Coleoptera_incl_Larvae	0.022	0.1045	0.211	∞	0.833
	Diplopoda	0.081	0.1267	0.639	∞	0.523
	Isopoda	-0.5548	0.1352	-4.104	∞	**<0.001**
	Lumbricidae	0.0227	0.1021	0.222	∞	0.824
Southern	Araneae	0.0744	0.1008	0.739	∞	0.460
	Chilopoda	-0.1756	0.0986	-1.781	∞	0.075
	Coleoptera_incl_Larvae	0.2055	0.0976	2.106	∞	**0.035**
	Diplopoda	-0.0072	0.1238	-0.058	∞	0.954
	Isopoda	-0.1131	0.1059	-1.069	∞	0.285
	Lumbricidae	-0.4059	0.1221	-3.325	∞	**0.001**

Table S8: Effects of silvicultural management intensity (standardized SMI) on macrofauna species richness by taxon and region, based on generalized linear mixed-effects models (GLMMs) with a negative binomial distribution. The table presents estimated effects (β values) of SMI on species richness per soil core, together with standard errors (SE), Wald z-ratios, asymptotic degrees of freedom (df), and p-values. Trends were extracted using the emtrends() function from the emmeans package, accounting for model interactions. Degrees of freedom are asymptotically infinite (∞) due to the use of Wald z-tests. Full model results are provided in Table S3. Estimated trends are shown in Figure S14.

Region	Taxon	Effect of SMI (standardized)	SE	z-ratio	Df	p-value
**Northern**	Lumbricidae	-0.373	0.1613	-2.313	∞	**0.021**
	Chilopoda	-0.1552	0.1757	-0.883	∞	0.377
	Diplopoda	0.2862	0.2412	1.186	∞	0.235
	Isopoda	0.636	0.4318	1.473	∞	0.141
**Central**	Lumbricidae	0.0669	0.0648	1.032	∞	0.302
	Chilopoda	-0.1535	0.0663	-2.316	∞	**0.021**
	Diplopoda	-0.0387	0.1033	-0.375	∞	0.708
	Isopoda	-0.248	0.1281	-1.937	∞	0.053
**Southern**	Lumbricidae	-0.2093	0.0796	-2.629	∞	**0.009**
	Chilopoda	-0.1014	0.0605	-1.676	∞	0.094
	Diplopoda	0.1028	0.0963	1.067	∞	0.286
	Isopoda	-0.1226	0.1163	-1.055	∞	0.292

Table S9: Results of linear models on variations in meso- and macrofauna stability (inverse coefficient of variation) as affected by the factors taxon (Oribatida, Collembola, Mesostigmata, Lumbricidae, Isopoda, Diplopoda, Chilopoda, Araneae, Coleoptera), region (northern, central, southern), forest management intensity (Sylvicultural Management Index – SMI), precipitation in the winter before (precipitation winter) or at the time of sampling (precipitation spring).

	**Mesofauna stability**				**Macrofauna stability**			
Factor	Sum of squares	df	F-value	p-value	Sum of squares	df	F-value	p-value
**Taxon**	0.04	2	0.05	0.947	21.91	5	23.93	**<0.001**
**Region**	3.46	2	4.50	**0.013**	5.47	2	14.92	**<0.001**
SMI	0.48	1	1.24	0.267	0.37	1	2.03	0.156
Precipitation winter	0.21	1	0.56	0.457	0.11	1	0.60	0.441
Precipitation spring	0.03	1	0.08	0.778	0.00	1	0.03	0.869
**Taxon x Region**	3.32	4	2.16	0.078	3.56	10	1.94	**0.040**
Taxon x SMI	0.45	2	0.59	0.558	1.04	5	1.13	0.343
**Region x Precipitation winter**	4.07	2	5.28	**0.006**	0.58	2	1.57	0.209
Region x Precipitation spring	0.18	2	0.23	0.794	0.25	2	0.68	0.507
Region x SMI	1.17	2	1.52	0.223	0.19	2	0.52	0.592
Taxon x Region x SMI	1.05	4	0.68	0.606	2.10	10	1.15	0.329
Residuals	46.19	120			42.50	232		

Table S10: Results of linear models on the influence of mean density, effective diversity, asynchrony (-η), management intensity (SMI – Sylvicultural Management Index) and region on the stability (inverse coefficient of variation) of mesofauna taxa (Oribatida, Collembola and Mesostigmata).

	**Oribatida stability**				**Collembola stability**				**Mesostigmata stability**			
Factor	Sum of squares	df	F-value	p-value	Sum of squares	df	F-value	p-value	Sum of squares	df	F-value	p-value
Density	0.03	1	0.31	0.581	0.04	1	0.64	0.430	0.14	1	2.73	0.108
**Effective Diversity**	1.37	1	16.44	**<0.001**	0.00	1	0.01	0.920	1.31	1	25.54	**<0.001**
**Asynchrony**	1.47	1	17.55	**<0.001**	0.84	1	12.56	**0.001**	2.85	1	55.85	**<0.001**
SMI	0.01	1	0.15	0.705	0.00	1	0.06	0.804	0.00	1	0.01	0.940
**Region**	1.02	2	6.11	**0.006**	1.06	2	7.90	**0.002**	0.15	2	1.47	0.244
Density x Region	0.21	2	1.28	0.291	0.21	2	1.58	0.220	0.19	2	1.90	0.165
Effective Diversity x Region	0.39	2	2.32	0.114	0.29	2	1.52	0.135	0.18	2	1.76	0.187
Asynchrony x Region	0.20	2	1.17	0.323	0.20	2	0.20	0.233	0.07	2	0.70	0.505
SMI x Region	0.30	2	1.77	0.186	0.03	2	0.20	0.820	0.10	2	0.98	0.385
Residuals	2.76	33			2.21	33			1.69	33		

Table S11: Results of linear models on the influence of density, effective diversity, asynchrony (-η) and management intensity (SMI – Sylvicultural Management Index) on the stability (inverse coefficient of variation) of macrofauna taxa (Lumbricidae and Chilopoda).

	**Lumbricidae stability**				**Chilopoda stability**			
Factor	Sum of squares	df	F-value	p-value	Sum of squares	df	F-value	p-value
Density	0.48	1	3.34	0.095	0.02	1	0.14	0.710
Effective Diversity	0.00	1	0	0.957	0.15	1	1.21	0.284
**Asynchrony**	0.08	1	0.54	0.478	2.13	1	16.91	**<0.001**
SMI	0.01	1	0.08	0.779	0.08	1	0.67	0.423
**Region**	0.46	2	1.62	0.242	0.89	2	3.54	**0.046**
Density x Region	0.05	2	0.18	0.836	0.06	2	0.22	0.805
Effective Diversity x Region	0.41	2	1.45	0.276	0.21	2	0.83	0.450
Asynchrony x Region	0.09	1	0.60	0.454	0.00	1	0.01	0.930
Residuals	1.57	11			2.78	22		

Table S12: Effects of winter precipitation on mesofauna stability by region, based on linear mixed-effects models (LMMs). The table presents estimated effects (β values) of standardized precipitation of the preceding winter on stability metrics, together with standard errors (SE), t-ratios, degrees of freedom (df), and p-values. Trends were extracted using the emtrends() function from the emmeans package, with all covariates held at reference levels. Bold values indicate statistically significant results ($p < 0.05$). Full model results are provided in Table S9. Estimated trends are shown in Figure S15.

Region	Effect of Precipitation in winter	SE	95% CI (β)	*t*	*df*	*p*-value
Northern	0.026	0.038	[–0.049, 0.101]	0.68	120	0.496
Central	0.224	0.090	[0.045, 0.403]	2.47	120	**0.015**
Southern	–0.074	0.037	[–0.147, –0.001]	–2.01	120	**0.047**

References

Anderson, J. P. E., and K. H. Domsch. 1978. "A physiological method for the quantitative measurement of microbial biomass in soils." *Soil Biology and Biochemistry* 10: 215-221.

Beck, T, R. G. Joergensen, E. Kandeler, F. Makeschin, E. Nuss, H.R. Oberholzer, and S. Scheu. 1997. "An inter-laboratory comparison of ten different ways of measuring soil microbial biomass C." *Soil Biology and Biochemistry* 29 (7): 1023-1032.

Fischer, M., O. Bossdorf, S. Gockel, F. Hänsel, A. Hemp, D. Hessenmöller, G. Korte, J. Nieschulze, S. Pfeiffer, D. Prati, S. Renner, I. Schöning, U. Schumacher, K. Wells, F. Buscot, E. K. V. Kalko, K. E. Linsenmair, E.-D. Schulze, and W. W. Weisser. 2010. "Implementing large-scale and long-term functional biodiversity research: The Biodiversity Exploratories." *Basic and Applied Ecology* 11: 473-485.

Kreklow, J., B. Tetzlaff, G. Kuhnt, and B. Burkhard. 2019. "A rainfall data intercomparison dataset of RADKLIM, RADOLAN, and rain gauge data for Germany." Data 4 (3): 118.

Natkhin, M., J. Steidl, O. Dietrich, R. Dannowski, and G. Lischeid. 2012. "Differentiating between climate effects and forest growth dynamics effects on decreasing groundwater recharge in a lowland region in Northeast Germany." *Journal of Hydrology* 448-449: 245-254.

Schall, P., and C. Ammer. 2013. "How to quantify forest management intensity in Central European forests." *European Journal of Forest Research* 132: 379-396.

---.2023. “SMI annual dynamics - Silvicultural management intensity dynamics on all forest EPs, 2008–2020, Version 9.” – Biodiversity Exploratories Information System. Dataset ID= 31217.

Scheu, S. 1992. "Automated measurement of the respiratory response of soil microcompartments: active microbial biomass in earthworm faeces." *Soil Biology and Biochemistry* 24: 1113-1118.
